# Molecular identity crisis: environmental DNA metabarcoding meets traditional taxonomy—assessing biodiversity and freshwater mussel populations (Unionidae) in Alabama

**DOI:** 10.7717/peerj.15127

**Published:** 2023-04-03

**Authors:** Laura L. Hauck, Carla L. Atkinson, Jessica A. Homyack, Brooke E. Penaluna, Clay Mangum, Ashley A. Coble, Jami Nettles, Jamie E. Thornton-Frost, Miranda J. Fix

**Affiliations:** 1 Pacific Northwest Research Station, U.S. Department of Agriculture, Forest Service, Corvallis, OR, USA; 2 Department of Biological Sciences, University of Alabama, Tuscaloosa, AL, USA; 3 Weyerhaeuser Company, Centralia, WA, USA; 4 Weyerhaeuser Company, De Queen, AR, USA; 5 National Council for Air and Stream Improvement (NCASI), Corvallis, OR, USA; 6 Weyerhaeuser Company, Columbus, MS, USA; 7 Weyerhaeuser Company, Seattle, WA, USA

**Keywords:** Unionidae, eDNA metagenomics, Biodiversity, Phylogenetic, Sipsey River, Pleurobema, Fusonaia, Mussel taxonomy

## Abstract

The use of environmental DNA (eDNA) to assess aquatic biodiversity is a growing field with great potential for monitoring and managing threatened species, like freshwater mussel (Unionidae) populations. Freshwater mussels are globally imperiled and serve essential roles in aquatic systems as a food source and as a natural water filter making their management essential for ecosystem health. Unfortunately, mussel populations are often understudied, and challenges exist to accurately and efficiently describe the full suite of species present. Multispecies eDNA approaches may also be more challenging where freshwater mussel populations are most diverse due to ongoing and significant taxonomic restructuring that has been further complicated by molecular phylogenies using mitochondrial genes. For this study, we developed a microfluidic metabarcoding array that targets a wide range of species, from invertebrates to fishes, with an emphasis on detecting unionid mussels known to be present in the Sipsey River, Alabama. We compared mussel species diversity across six sites with well-studied mussel assemblages using eDNA surveys and traditional quadrat surveys in 2016. We examined how factors such as mussel population density, biomass and location in the river substrate impacted our ability to detect certain species; and investigated unexpected eDNA detections through phylogenetic analysis. Our eDNA results for fish and mussel species were broadly consistent with the data from traditional electrofishing and quadrat-based field surveys, although both community eDNA and conventional sampling detected species unique to that method. Our phylogenetic analysis agreed with other studies that treat *Pleurobema decisum* and *P. chattanoogaense* as synonymous species; however, they are still listed as unique species in molecular databases which complicates their identity in a metabarcoding assay. We also found that *Fusconaia flava* and *F. cerina* are indistinguishable from one another using a portion of the NADH dehydrogenase Subunit 1 (ND1) marker, which may warrant further investigation into whether or not they are synonymous. Our results show that many factors impacted our ability to detect and correctly identify Unionidae mussel species. Here we describe the obstacles we faced, including the murky phylogeny of Unionidae mussels and turbid river conditions, and our development of a potentially impactful freshwater mussel monitoring eDNA assay.

## Introduction

Effective solutions for conserving biological diversity depend on a comprehensive understanding of the full suite of species present in a geographic area. In locations where biodiversity is high but relatively understudied, challenges exist to accurately and efficiently describe the full suite of species present and identify potential cryptic species. The southeastern United States is a center of global biodiversity for freshwater species and a region where the conservation status of many species is jeopardized, species distribution boundaries are not well-understood, and conservation funding is low compared to other regions (Haag & Williams, 2013; Richman et al., 2015; Elkins et al., 2019). Thus, developing techniques to rapidly, efficiently, and accurately quantify occupancy for a broad range of aquatic species across space and time is greatly needed for proactive progress towards conservation and monitoring goals.

Freshwater mussels are a diverse group in aquatic systems of the southeastern United States, with the majority of North American species occurring in rivers. However, 65% of North American mussel species are at risk due to historical and current degradation of habitat from anthropogenic activities that increase sediment in water bodies, create impoundments, facilitate invasive competitors, and other factors (Haag & Williams, 2013). Mussels have a complex life history, where reproductive success is dependent on one or more host fish species for attachment of a larval stage (glochidia) to gills or other body parts before detaching and settling on or in the water’s substrate (Haag, 2012). Consequently, mussels also are sensitive to factors that negatively impact host fish. Mussels serve several ecological roles by improving water quality by filter feeding, contributing to nutrient cycling, serving as prey for numerous other species, and by stabilizing substrates (Atkinson et al., 2013; Atkinson et al., 2018; Malmqvist, Wotton & Zhang, 2001; Vaughn & Hakenkamp, 2001). Despite urgency due to severe declines and extinctions of freshwater mussels and known ecological benefits of conservation, knowledge of species diversity across and within river systems can be time-consuming to assess. Further, phylogenetic relationships and species descriptions are changing, as many freshwater mussel species can be difficult to distinguish based on morphological characteristics alone, which can be influenced by environmental factors (Turgeon et al., 1998; Haag, 2012; Williams et al., 2017). In addition, DNA barcoding studies are discovering species that were previously thought to be extinct (Campbell et al., 2008), which may be an indication of species synonyms or hidden species strongholds and in both cases is revealing populations in need of protection.

The use of environmental DNA (hereafter eDNA) as a tool to detect presence of species in freshwater environments has grown rapidly with most studies examining endangered or invasive fishes (Belle, Stoeckle & Geist, 2019; Coble et al., 2019). The non-invasive approach of filtering water samples and analyzing fragments of shed DNA has many benefits, such as ease of collection of samples, ability to have relatively high detection rates for rare species and sampling many species with one approach (Coble et al., 2019). Use of eDNA has also expanded from single species detections to multiple species metabarcoding arrays that can capture a biodiversity snapshot at a given location (Valentini et al., 2016; Hauck et al., 2019). These applications can serve as a repeatable and standardized tool for documenting range shifts, presence of single or multiple species, relative abundance among species communities (Hauck et al., 2019) and population genetics (Weitemier et al., 2021). Strength of detections with eDNA and DNA degradation can vary with environmental factors (*e.g*., streamflow, water temperature, target species, substrate), and challenges with false positives exist. Freshwater mussels may also have unique eDNA sampling challenges because many species have cryptic life stages (*e.g*., microscopic larvae in suspension, adults buried in substrate) whose availability for detection may vary seasonally. Improving our knowledge on effective deployment of eDNA as a sampling technique requires direct comparisons to traditional field methods, particularly for taxa like freshwater mussels that need novel methods to address challenges from imperilment and limited funding for conservation.

Existing validation studies using eDNA to detect occurrences of both native and non-native mussels primarily have used single species *in situ* approaches across known or manipulated densities of freshwater mussels (*e.g*., Shogren et al., 2019; Gasparini et al., 2020). Fewer freshwater mussel studies have used metabarcoding methods, which can detect multiple species with a single sample, while also identifying other taxonomic groups including host fishes. Several recent studies have demonstrated the utility of metabarcoding to describe and compare mussel diversity in riverine systems, although some challenges remain (Coghlan et al., 2021; Klymus et al., 2021; Prié et al., 2021; Marshall et al., 2022). For this study, we developed a microfluidic metabarcoding array that targets a wide range of species that are known to be present in a diverse southeastern USA stream, the Sipsey River, Alabama. This array was developed with an emphasis on capturing DNA signal from freshwater mussel assemblages (including rare and/or cryptic mussel species) and fishes, some of which are potential hosts for mussels; but we also targeted, with varying success, amphibians, aquatic insects, turtles, waterborne pathogens (such as *Saprolegnia* and *Phytophthora*), and crayfish.

We capitalized on a unique opportunity to advance our understanding of sampling techniques for freshwater mussel assemblages on a river system with a diverse and intact Unionidae mussel community. We compared detections using eDNA to estimates from traditional quadrat sampling of freshwater mussels at six locations along a 44.9 km stretch of the Sipsey River in western Alabama, USA. The Sipsey River is a 2,044 km^2^ alluvial river watershed with extensive forested floodplain wetlands that include secondary channels and oxbow lakes (McGregor & O’Neil, 1992). Historical and current human impacts along the length of the river include impoundments at the southern end, forest harvesting of commercial pine plantations in adjacent uplands in the middle section, and coal mining in the headwaters. Compared to other similar river systems, however, the Sipsey River is relatively unmodified by human activities. Consequently, it has low background nutrient concentrations (Atkinson et al., 2019) and one of most diverse and abundant freshwater biological communities in the southeastern United States (McGregor & O’Neil, 1992) including most of its historical native mussel fauna (Haag & Warren, 2010). The Sipsey River was an ideal location to compare mussel diversity and occupancy estimates from traditional and eDNA methods due to the known high aquatic biodiversity estimates and fine-scale information on mussels from long-term sampling (Haag & Warren, 2010).

Here, we evaluate the benefits and limitations of eDNA as a rapidly evolving technique to document the presence of the entire mussel community and assess the species consortium present in the river. Our aim is to improve methodology of metabarcoding in different field conditions and compare our eDNA detections with extensive field sampling of the Unionidae taxonomic group. We also compared the assemblage of potential fish hosts detected by eDNA with an Alabama Geological Survey field collection conducted 2011–2013 (Bearden et al., 2019). We predicted that metabarcoding would be an effective method to detect the presence of a diverse array of species as compared to traditional survey approaches, but that environmental conditions of our study system would present challenges to laboratory analyses.

## Materials and Methods

### Study area

**Figure 1:**
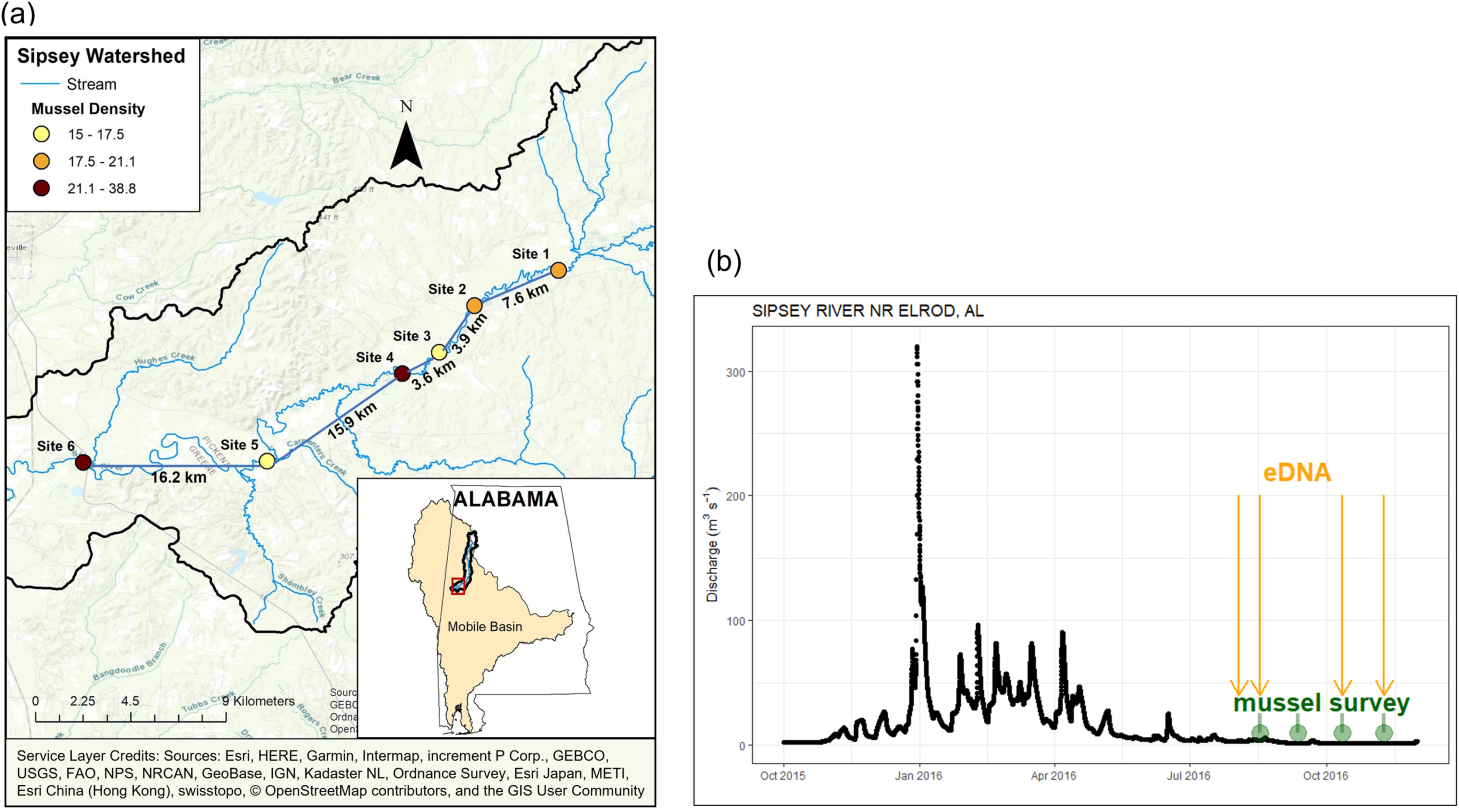
Location and discharge parameters of six study sites along Sipsey River, Alabama USA. (A) Distances reflect euclidean river distances between sites and mussel density (individuals/m^2^) is color coded for each site. (B) Streamflow discharge (m^3^s^−1^) in Sipsey River near Elrod, Alabama (USGS 02446500). Dates for traditional mussel survey (green circles) and water collection for eDNA extraction (yellow arrows) are shown. Service Layer Credits: Sources: Esri, HERE, Garmin, Intermap, increment P Corp., GEBCO, USGS, FAO, NPS, NRCAN, GeoBase, IGN, Kadaster NL, Ordnance Survey, Esri Japan, METI, Esri China (Hong Kong), swisstopo, © OpenStreetMap contributors, and the GIS User Community.

We selected six sites on the Sipsey River (Fig. 1) where freshwater mussels had been sampled previously (Pierson, 1991; Haag & Warren, 2010; Atkinson & Forshay, 2022). In addition to sampling mussels with eDNA and traditional methods, we quantified environmental characteristics of the study area. We derived watershed areas for each sampling point using the Spatial Analyst Toolkit in ArcMap 10.6 (Environmental System Research Institute, Redlands, CA, USA) with a 30-m digital elevation model (DEM) from the National Elevation Dataset. We obtained land cover (30-m resolution) for the United States from the 2011 National Land Cover Database. We measured water temperature, pH, and conductivity with a YSI multiparameter probe. In addition, we filtered water samples through a Gelman A/E, GFF, 0.7-μm nominal pore size to determine water chemistry and placed them on ice. We determined NH_4_-N and soluble reactive phosphorus (SRP) with a Lachat Quikchem 8000 flow-injection auto-analyzer using colorimetric methods (Lachat Instruments, Milwaukee, MN, USA). See Table 1 for a summary of study site characteristics.

**Table 1:** Study site characteristics from upstream to downstream locations within the Sipsey River, Alabama. River distance represents distance from headwaters. Land use characteristics were determined from the National Land Cover Database for the catchment. We present mean background nutrient concentrations for ammonium (NH4-N) and soluble reactive phosphorus (SRP).

Site name	Date of quadrat survey	River distance (km)	Catchment size (km^2^)	Developed (%)	Forest (%)	Crop (%)	Wetland (%)	NH_4_-N (µg L^−1^)	SRP (µg L^−1^)	Mean stream width (m)
Site 1 Wendell 6	12-Sept-2016	227.4	1,636	4.49	71.86	7.54	15.40	24.3 ± 1.0	7.3 ± 1.9	22.0
Site 2 Wendell 5	19-Sept-2016	234.2	1,707	4.44	71.74	7.56	15.54	19.6 ± 2.3	5.2 ± 0.7	23.7
Site 3 Wendell 3	30-Sept-2016	237.7	1,738	4.40	71.70	7.50	15.69	21.1 ± 0.6	4.8 ± 0.4	18.2
Site 4 Mussel Mania	11-Aug-2016	240.8	1,752	4.40	71.68	7.50	15.71	11.9 ± 4.0	3.0 ± 1.1	26.8
Site 5 Wendell 2	28-July 2016	254.9	1,871	4.27	71.84	7.50	15.70	18.5 ± 0.3	5.8 ± 0.5	20.8
Site 6 Station 5	30-Aug-2016	272.3	1,991	4.16	72.00	7.34	15.81	16.0 ± 1.7	4.9 ± 0.5	29.6

### Traditional surveys

We conducted quantitative mussel surveys in 60–80 m reaches between July-October 2016 across the six sampling sites. We first visually determined the extent of each mussel aggregation by snorkeling and then divided each total reach into 20 m sections (3–4 sections per site) in which three random numbers were selected to determine our transects. Transects were run perpendicular to stream flow within each 20 m section. Across each transect we excavated the sediment from a 0.25 m^2^ quadrat to a depth of 15 cm every 1.5 m (6–20 quadrats per transect) retaining all the material dug into a mesh bag. To determine burial preference, prior to excavating the quadrat we lightly moved our hand over the sediment surface and recovered mussels that appeared at the surface and were not completely buried and placed them into a separate small bag that was placed within the quadrat’s mesh bag. Each bag was individually labeled, and all mussels were sorted through a series of sieves with species identification (Williams, Bogan & Garner, 2008) and lengths recorded for each individual. This sampling regime resulted in us sampling approximately 2.5% of the total area of the designated reach and we determined mussel species richness, density (indiv/m^2^), and estimated biomass using published length-dry mass relationships (see Atkinson et al., 2020) at each site.

We used fish community data collected by the Geological Survey of Alabama (GSA) and the Alabama Department of Environmental Management (ADEM) in 2011–2013 in the Sipsey River and major tributaries to evaluate fish species composition and abundance. Sampling combined seining and backpack electrofishing following the methodology of the Index of Biologic Integrity in the Hills and Coastal Terraces Ichthyoregion in Alabama (O’Neil & Shepard, 2011, Bearden et al., 2019). Briefly, surveyors performed a minimum of ten sampling efforts at each distinct type of habitat; riffle, pool, run or shoreline at each site. Freshwater mussel collection was conducted under US Fish and Wildlife Service permit #TE68616B-1 and Alabama Department of Conservation and Natural Resources permit #2016077745468680 to C.L. Atkinson.

### Water sampling and eDNA extraction

We collected water samples for eDNA analysis at the six sampling sites on four occasions from August to November 2016 when we expected flow and turbidity to be low and when we hypothesized that eDNA would be most concentrated. We collected 4, 1-L samples of water at each site and sampled all sites in a day from downstream to upstream to avoid contamination. We repeated this sampling four times with each occasion separated by >2 weeks: August 3, August 17, October 12, and November 9, 2016. Conducting repeated visits separated by time allowed us to capture eDNA across a range of environmental conditions and through life history milestones (*e.g*., reproductive periods) of some of the target Unionidae community. This information is key to quantifying conditions that influence detection of mussel species from eDNA-based surveys (Schmidt et al., 2013).

Some sites were accessible on foot and others required a short canoe paddle. To prevent cross-site contamination, we entered sites from a downstream direction, and we decontaminated equipment (*e.g*., bottles, tweezers, waders) with a 50% bleach solution. With a gloved hand, each bottle was labeled, opened, and filled in the river at arm’s length from the shore, downstream but as close to the mussel quadrat surveys as possible, with the 1-L bottle mouth facing upstream in the center of the water column. Water depth at each site varied from 16 cm to 60 cm so the collection was anywhere from approximately 8 cm to 30 cm above the benthos. Water samples were kept in coolers on wet ice for transport back to the lab, where they were placed in a standard refrigerator until filtering.

We filtered all four samples collected at each site and date with Cellulose Acetate filters but varied the pore size such that two samples were filtered with 3 µm and two with 5 µm pore size for each collection. Methods to filter water samples in the field were not feasible in our study area because of the high levels of turbidity that characterize many Gulf Coast river systems. Instead, we developed a vacuum pump manifold system set to 20 PSI in a laboratory setting to filter water samples within 24 h of collection to minimize degradation of DNA. Numerous precautions were incorporated into laboratory practices to minimize risk of contamination between samples, including using new gloves between samples, and cleaning equipment and the workstation with bleach prior to filtering and between samples. A negative control water sample was also run through the filtering process and included on the array. After vacuum filtration, each filter was carefully rolled with a gloved hand and placed in a labeled and sealed vial and frozen prior to shipment on dry ice to the United States Department of Agriculture (U.S.D.A) Forest Service Pacific Northwest Research Station in Corvallis, Oregon for DNA extraction and subsequent analysis. We conducted pilot tests on other nitrocellulose filter pore sizes of 0.45 µm (Hauck et al., 2019) and 1.2 µm but found that both were too fine to allow filtering of water from our study area to be completed within 24 h. Even with the vacuum pump, the turbid water samples clogged the filters and stopped the filtering process. DNA was extracted and processed as described in Hauck et al. (2019). Briefly, the eDNA was extracted using the inhibitor removing Power Water DNA extraction kit (Qiagen, Hilden, Germany) per manufacturer’s instructions and the samples were cleaned and concentrated post-extraction with the Zymoclean© Large Fragment DNA Recovery Kit (Zymo Research, Irvine, CA, USA).

### Microfluidic PCR amplification and massively parallel sequencing

Microfluidic high throughput PCR amplification of the eDNA was performed on the Fluidigm 48.48 Access Array™ (Fluidigm, San Francisco, CA, USA; Fluidigm Corporation, 2016) as previously described in Hauck et al. (2019). Specifically, the Access Array uses integrated fluidic circuits and a 4-primer amplicon tagging scheme in which target-specific primer pairs amplify 48 different targets in combination with sample-specific barcoded primer pairs in 48 different samples allowing for the simultaneous amplification of barcoded targets in each of the 2,304 individual reaction chambers on each array. Many of the taxon-general universal metabarcoding primers we used in Hauck et al. (2019) were designed to amplify diverse classes of organisms (*e.g*., ray-finned fishes (Teleostei) and amphibians (Batrachia), Chondrostei fish, mussels (Bivalvia), and insects), by targeting the 12S rDNA region used by Valentini et al. (2016), as well as 16S, 18S, and internal transcribed spacer (ITS) rDNA. Due to their universal nature, they were used again in this study. Additional primers were designed to target organisms found in the Gulf of Mexico coastal rivers. The new primers were developed as described in the previous study (Hauck et al., 2019), and targeted mostly freshwater mussels, crayfishes, warm water fish, and turtles using mitochondrial genes commonly used for eDNA metabarcoding purposes: cytochrome B (CytB), 12S and 16S ribosomal genes, Cytochrome Oxidase Subunit I (COI), NADH dehydrogenase Subunit 1 (ND1), subunit 2 (ND2), subunit 4 (ND4), and Subunit 5 (ND5). See Table S1 for all primer sequences and the associated species and gene targets used in this study. We determined previously through test PCR reactions and preliminary Fluidigm experiments that the optimal Fluidigm submission concentration for eDNA is 12–15 ng/µl; unfortunately, many of these samples did not contain enough DNA to be submitted at the optimal concentration. As a result, we submitted the DNA samples in the following categories: 12, 6, 3, 3 > 1, or <1 ng/µl (see Table 2 for final sample submission details).

**Table 2:** Total number of samples submitted by concentration for eDNA metabarcoding on six sites along the Sipsey River, Alabama, USA.

DNA concentration submitted (ng/ul):	12	6	3	3><1	1>	Total samples
Site 1	10	1	0	0	4	15
Site 2	8	0	0	4	4	16
Site 3	7	1	2	6	0	16
Site 4	9	0	0	4	3	16
Site 5	6	3	1	1	1	12
Site 6	8	5	1	2	0	16

The negative control consisted of samples N1R3 (plate 1) and N4R3 (plate 2), which are ultrapure water carried through the sample filtration process. Our positive controls followed the methods in our previous study (Appendix 1, Hauck et al., 2019) with the goal of 2 × 10^4^ molecules per amplicon per 31.35 nl volume (the size of a Fluidigm Access Array reaction, after accounting for primer and mastermix volume). Target species DNA was derived from organisms that were collected by field sampling, through other researchers, or from acquiring tissues from museum specimens. All samples were sent to the USDA Forest Service Pacific Northwest Research Station in Corvallis, Oregon for DNA extraction. Unfortunately, the resulting DNA was often degraded, particularly when derived from the museum samples which had been stored in formaldehyde for a significant amount of time. Amplification was still attempted, and a basic standard was composed of amplicons for those few species whose DNA we were able to generate amplicons from (see Table S2 for a list of positive control species). A modified positive was also added to one plate; it consisted of the basic standard spiked with the same copy number of amplicons generated for the metabarcoding primers used in both the previous study and this one.

DNA samples and primers were sent to the Roy J. Carver Biotechnology Center at the University of Illinois for amplification on their Fluidigm instrument and sequencing on their MiSeq instrument using 2X250 V2 chemistry. Amplification was done using a 2-step Fluidigm cycling protocol and a modified template-specific annealing temperature to 58 °C. Bovine serum albumin (BSA) was added at 0.2 μg/μl to alleviate PCR inhibition from contaminants in environmental DNA (Romanowski, Lorenz & Wackernagel, 1993; Widmer, Seidler & Watrud, 1996) and to mitigate inhibitor-driven bias (Valentini et al., 2016). Samples were barcoded with 10 basepair (bp) indices during amplification and then pooled for gel cleanup and size selection prior to sequencing. All raw sequence reads from this dataset are accessible to the public, they have been uploaded into the Sequence Read Archive (SRA) at the National Center for Biotechnology Information (NCBI) and can be accessed by searching for BioProject Accession Number PRJNA902311.

### Bioinformatic analysis

Sequences were returned from the Roy J. Carver Biotechnology Center demultiplexed by index and by primer using a custom pipeline they designed to sort Fluidigm data. For this run, they allowed one mismatch in the index, and two mismatches in the primer. Due to anomalies found in the read two data during joining of the paired-end sequences, only ‘read 1’ sequences were used in subsequent analyses. Trimming of the ‘read 1’ sequences was done in primer groups using Trimmomatic v. 0.33 (Bolger, Lohse & Usadel, 2014). Using amplicon specific information, the 5’ end of the read was trimmed the length of the primer plus five nucleotides and the overall length of the read was cut off at 200 bp to avoid analyzing the 3’ end of the sequence where quality scoring of read one sequencing began to drop off. The taxa assigning pipeline that we used for this dataset is KMA version 1.2.23, which uses a kmer-based conclave scoring method (Clausen, Aarestrup & Lund, 2018). Once run through KMA, the data was then piped through CC-Metagen version 1.2.2 (Marcelino et al., 2020) for the final assignment of taxonomy to the read. The CC-Metagen pipeline was originally developed to assess gut microbiome and fungal populations using singular, short reads with high conservation within species. Because our data consists of many different gene targets of varying conservation, length, and taxonomic origin, we needed to modify the thresholds to better capture species richness. We found the following settings best fit this particular data set: class = 79.0, order = 80.0, family = 84.0, genus = 91.4, and species = 94.4. Any reads showing up in the negative control may be the result of index hopping, sequencing error in the indices or contamination. We used the negative control to calculate a 95th percentile minimum read threshold. Any reads at or below this threshold were removed from the dataset. Counts above the threshold were considered “positive hits” for that taxon in that sample × primer combination. A Krona chart for hierarchical viewing of all counts above the threshold for all sample sites was generated using a krona excel template (Ondov, Bergman & Phillippy, 2011).

### Phylogenetic analysis

Based on the classification results of our eDNA data we decided to investigate the reads from unexpected species detections. Species assignments of *Fusonaia cerina* and *F. flava* and *Pleurobema decisum* and *P. chatanoogaense*, and *Elliptio sp*. eDNA reads from our dataset were investigated through phylogenetic analysis at the mitochondrial ND1 gene. ND1 sequences in our dataset were derived from multiple primers targeting different, and sometimes overlapping, fragments of ND1 ranging from 99 bp to 220 bp. Our reads reflect consensus sequences pulled from KMA alignments, and they are named by sample and preliminary KMA alignment classification. All available NCBI reference sequences for *P. decisum* (four), *P. chatanoogaense* (two) and *F. cerina* (seven) were downloaded for inclusion in alignments. *F. flava* had a very large number of sequences in NCBI, so seven representative sequences were selected with a preference for vouchered specimens. In addition, to provide an outgroup and guide tree branching, up to six representative sequences were pulled from NCBI for the following taxonomic groups within Ambleminae: *Quadrula* (outgroup), *Obovaria*, *Lampsilis* and *Reginaia*.

All sequence alignments and trees were created using Geneious® software version 10.2.6 created by Biomatters (available from https://www.geneious.com). Alignments were conducted using MUSCLE with a maximum number of 100 iterations with first iteration kmer4_6, and terminal gaps penalties were turned off to accommodate multiple sequence lengths in the alignment. Prior to conducting an alignment our consensus sequences were screened for residual primer or adapter sequence and trimmed if necessary. Maximum likelihood trees were drawn from the alignments using RAxML 8.2.11 with the nucleotide model GTR CAT I, which includes an estimate of proportion of invariable sites. The tree algorithm used was ‘Rapid Bootstrapping’ and ‘search for best-scoring ML tree’ with 1,000 random replicates to generate the final tree and bootstrap values. The tree was rooted with the Quadrula sequences. As unionid mussels of the Quadrulini tribe, not the Pleurobimini tribe we were investigating, they are a valid outgroup for tree rooting.

### Data analysis

We used summary statistics and graphics to discuss observed differences in species detection between our two sampling methods. This was necessary due to the dissimilar structures of the data sets themselves, the relatively small sample size of our data, and differences in the timing of data collection between the traditional samples and the eDNA samples. Traditional survey data was summarized to the site and species level. Our six sites used different numbers of quadrats during sampling (range 103 at Site 3 to 206 at Site 6) as a result of the differences in stream width and mussel aggregation length; therefore the total number of individual mussels encountered as well as total biomass was normalized to a per m^2^ value. We summarized eDNA results by site when comparing the eDNA detections directly to the traditional survey results. We also summarized the eDNA data by sample, replicate and filter size to evaluate the total number of unique taxonomic identities detected and the number of reads for each sample; and did comparisons on the amount of DNA submitted (as a categorical variable) to evaluate the impact of DNA quantity on the total number of reads and unique taxonomic identities returned.

## Results

### Mussel diversity and density from traditional quadrat surveys

**Figure 2:**
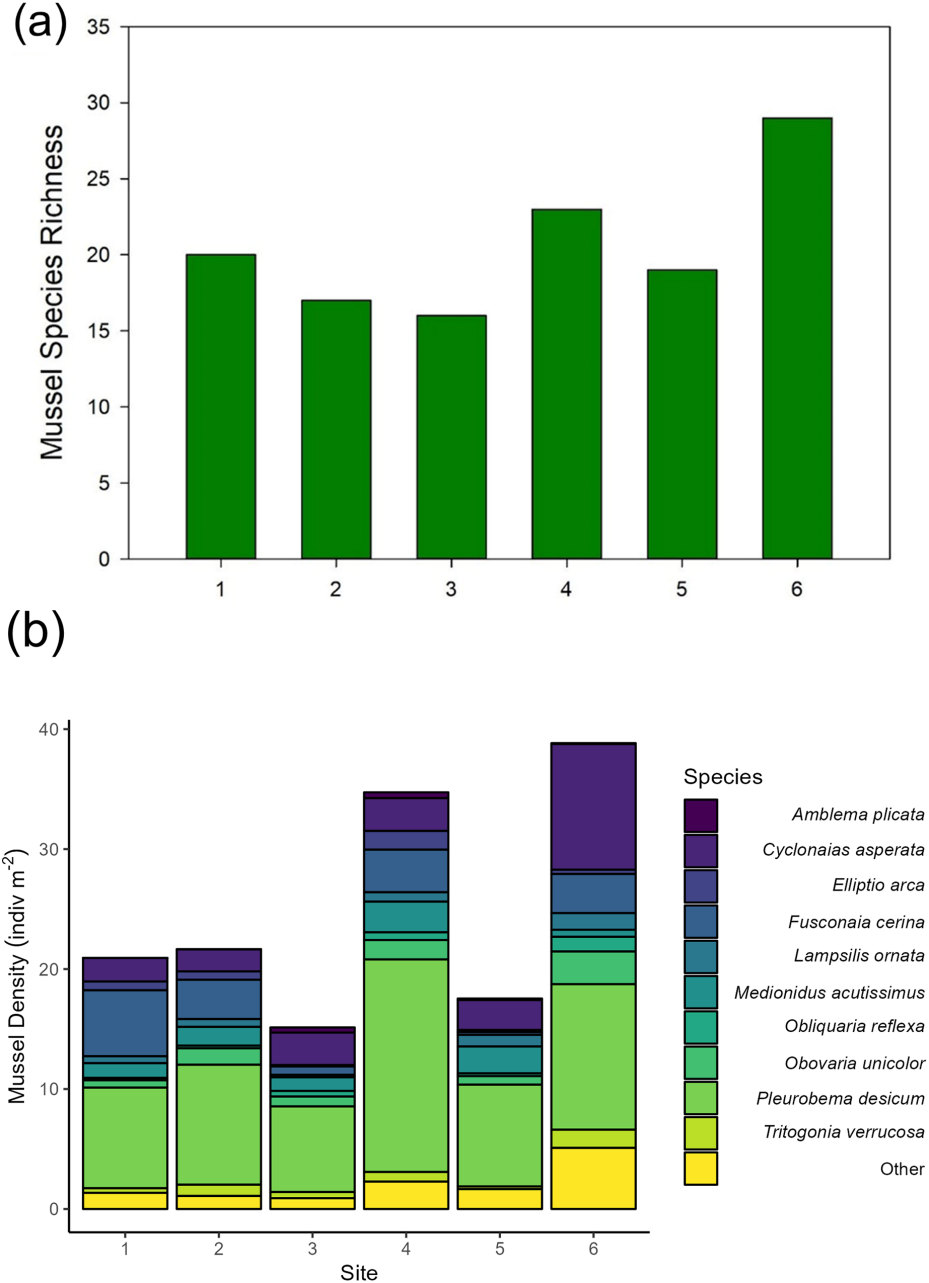
(A) Mussel species richness and (B) density of freshwater mussels determined from traditional quadrat survey at six sites on the Sipsey River, Alabama, USA.

Across our six sites, we sampled 779 quadrats and measured 6,195 individual unionid mussels in 2016, observing 17–29 species per site (Fig. 2). Unionid community densities (including all species) ranged 15.1–38.8 individuals per m^2^ and community biomass ranged 6.6–15.9 g per m^2^. The most abundant species encountered were *Pleurobema decisum*, *Cyclonaias asperata*, and *Fusconaia cerina*, respectively (Fig. 2).

### eDNA yield and metabarcoding results

The yields from eDNA extractions were rather low, and despite having larger pore sizes, the 5 µm filters consistently yielded more DNA than the 3 µm filters. Due to the poor yields, Site 3 is missing one of the 3 µm filter replicates for each collection time and is underrepresented in the overall eDNA pool. Site 1 is also missing one of the 3 µm filter replicates for the fourth collection point but is fully represented at the other timepoints. In addition, many samples were submitted below the optimal concentration of 12–15 ng/µl (Table 2). This likely led to increased missingness in our dataset and will require trouble shooting for future eDNA studies in turbid water systems.

Amplification of the standard positive control failed, and limited amplifications occurred in the modified positive control. This limits our ability to define false negative results. The only primer pair that produced reads in the negative control was a highly efficient pair targeting *Phytophthora*, a genus of plant pathogens. The calculated minimum threshold was five reads. To avoid false positives any primer x taxon x sample combination that had five or fewer total reads was dropped from the data set. After processing, 1.76 million reads were classified and included in the data set (1.67 million reads with controls removed). The KMA/CC-Metagen pipeline for classifying organisms yielded very few unexpected results. Of those classified reads, only 57 reads were identified as non-native fish in the genus *Oncorhynchus*, and 714 reads classified to salamanders found only in the Pacific Northwest. The rest of the reads were classified as organisms, or closely related organisms, that we would expect to find in a Southeastern US river. The source of the salamander reads likely resulted from barcode swapping or sorting errors with another array that was run concurrently. Since there were no reads from these species in the negative control, contamination was unlikely. We removed the salamander reads from the data set.

**Figure 3:**
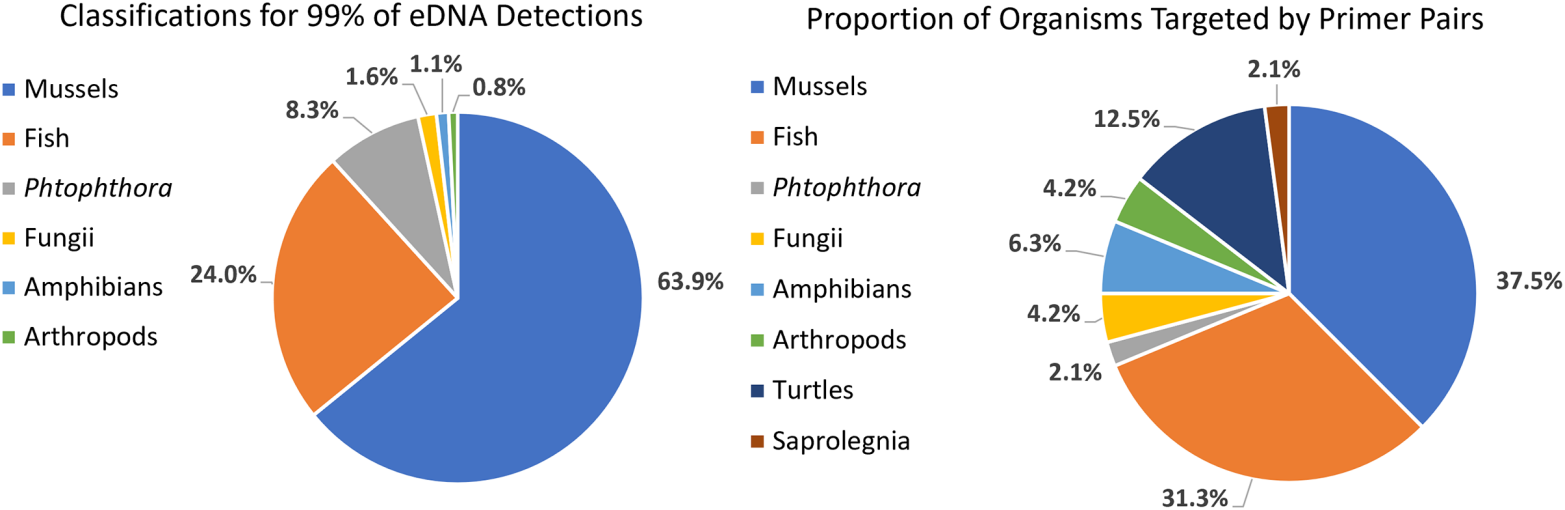
Targeting strategy impact on biodiversity capture from eDNA metabarcoding from samples collected on six sites along the Sipsey River, Alabama, USA.

**Figure 4:**
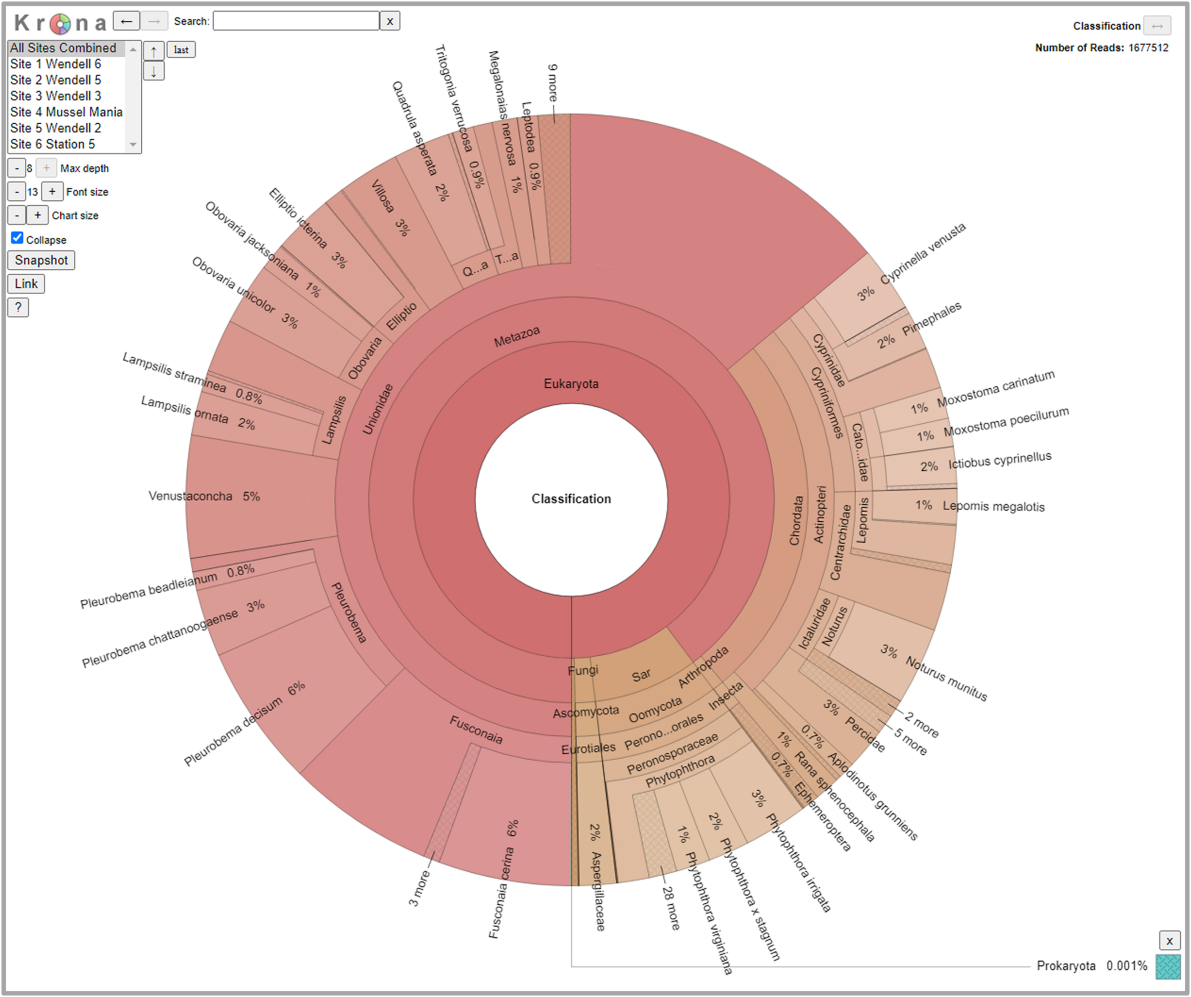
Static Image of a Krona Graph displaying a summary of all classifications from eDNA collected from all six sites along the Sipsey River, Alabama, USA. See File S1 for the interactive Krona Graph where the results can be explored by site and taxonomic grouping.

We detected 126 different species with this array, including taxa representing unionid mussels, fish, *Phytophthora*, fungi, amphibians, arthropods (insects and crustaceans), cyanophora, oomycota, apicomplexia, and bacteria. Additional detections were only taxonomically classified to family (21), genus (33) or order (2). We did not detect any turtles despite targeting them directly with six primer pairs and known occurrences of numerous species of freshwater turtles in the watershed. Not surprisingly, when all detections are summed, 99.6% of the reads are composed of six taxonomic groups. Of the 48 primer pairs that we deployed on the array, 18 targeted mussels and 15 targeted fish, which are the two taxonomic groups that we detected with the greatest number of reads (Fig. 3). The single *Phytophthora* primer pair captured the next highest number of reads. All read classifications summarized by site can be viewed in an interactive Krona Graph (File S1), see Fig. 4 to view a static image of the Krona Graph displaying a summary of all classifications for all sites.

**Figure 5:**
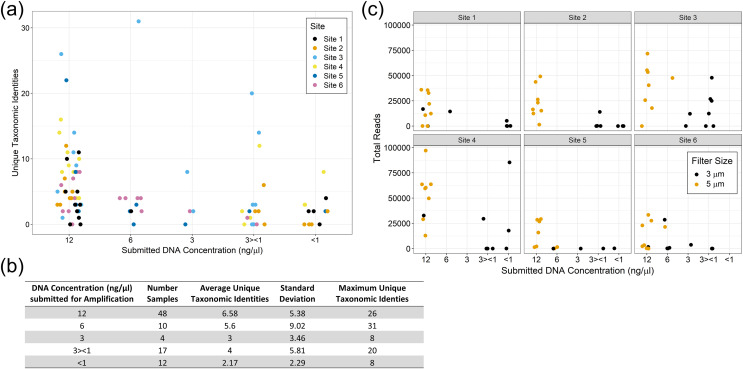
eDNA yields and performance of eDNA during amplification by submission concentration. Samples submitted at the optimal concentration of 12 ng/µl returned more unique taxonomic identities on average, however the results were highly variable (A) and (B). The filter pore size used during sample filtration impacted the yield per sample with the 5 µm filters producing more eDNA and returning more classified reads overall (C).

We did not observe strong patterns for DNA yield by site. There was a high variability between samples and overall number of reads that made it through the classification pipeline. This included numerous samples submitted at high concentration returning a low number of reads, and low concentration samples returning a relatively high number of reads. This may be a reflection of the overall post-extraction quality of the eDNA sample. We observed that samples filtered using the 5 µm filter often resulted in more DNA for submission and tended to have higher classified read returns (Fig. 5). The number of classified reads per sample ranged from 0 to 96,996 with an average of 18,442; and the number of unique taxonomic identities per sample ranged from 0 to 31 with an average of 5.25. The average number of unique taxonomic identities coming from the samples submitted at the optimal concentration of 12 ng/µl was 6.58, which was higher than the average overall (Fig. 5). Samples submitted at less than 1 ng/µl underperformed on average.

### Comparison between traditional surveys and eDNA detections

#### Fish

**Figure 6:**
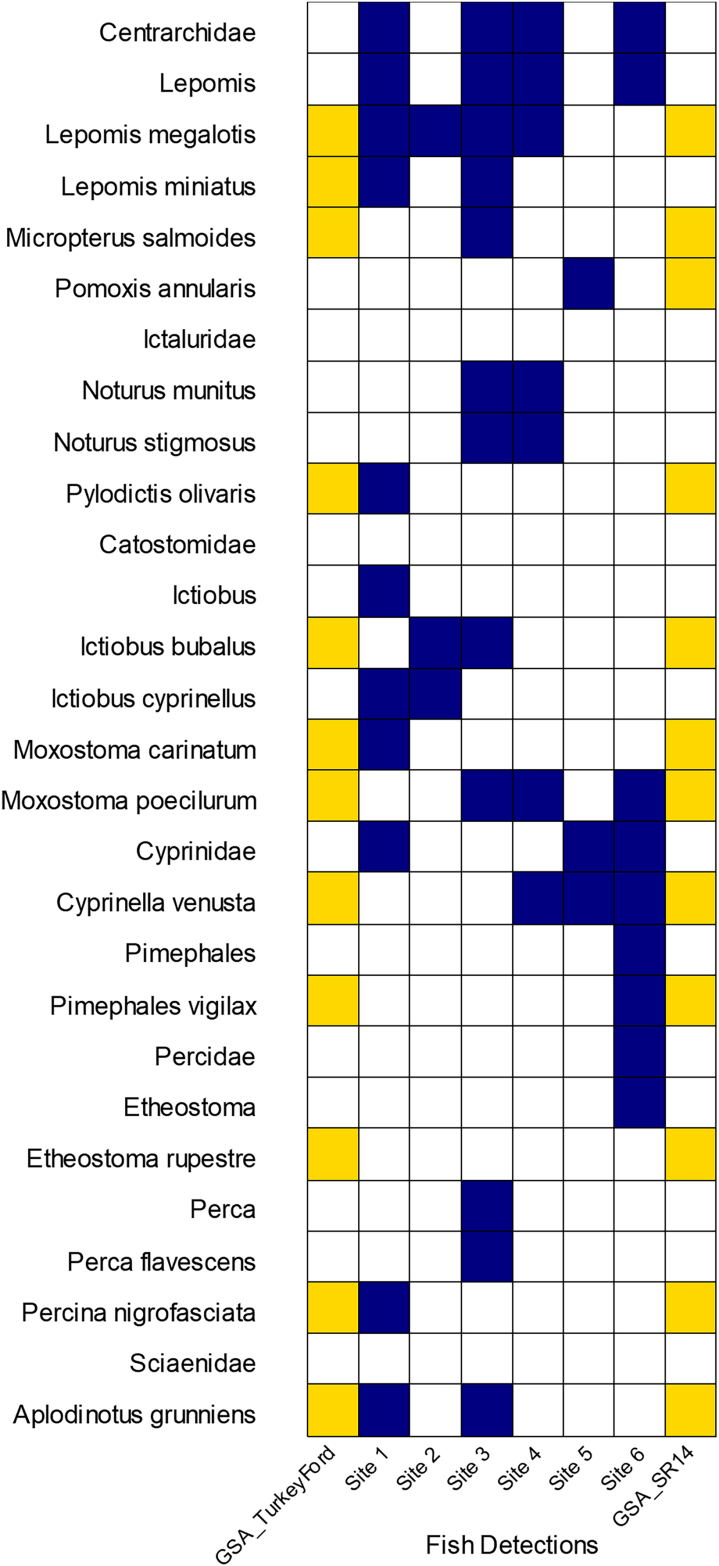
Comparison of GSA fish survey results to eDNA detections on seven sites along the Sipsey River, Alabama, USA. Yellow boxes indicate electrofishing detections from GSA survey, dark blue boxes indicate our eDNA detections.

We had 402,493 reads that classified as actinopteri with 26 species and nine genus level detections representing eight different families of ray-finned fishes. Of those reads, approximately 70% originated from universal primers that targeted the highly conserved 12S or 16S regions. These universal primers led to some classification issues where most of those reads were classified to family or genus but not to species. Most of the detections came from the following fish families: Cyprinidae, Centrarchidae, Ictaluridae, and Percidae. We loosely compared some of our fish detection results from eDNA to traditional surveys conducted 3–7 years prior by the Alabama Geological Survey (GSA), 2011–2013 (Bearden et al., 2019). The GSA survey occurred at two sites at or near to our study area. Our Site 6 corresponds with GSA Site SR14, separated only by a bridge. GSA Site Turkey Ford was approximately 4 km upstream from Site 1. Traditional surveys identified the Rock Darter *Etheostoma rupestre* in the Sipsey River whereas eDNA made a detection at the genus level for *Etheostoma*. Yellow Perch *Perca flavescens*, Bigmouth Buffalo *Ictiobus cyprinellus*, and two madtoms (*Notorus munitus* and *N. stifmosus*) were detected with eDNA, but not captured earlier in the traditional surveys (Fig. 6).

#### Mussels

**Figure 7:**
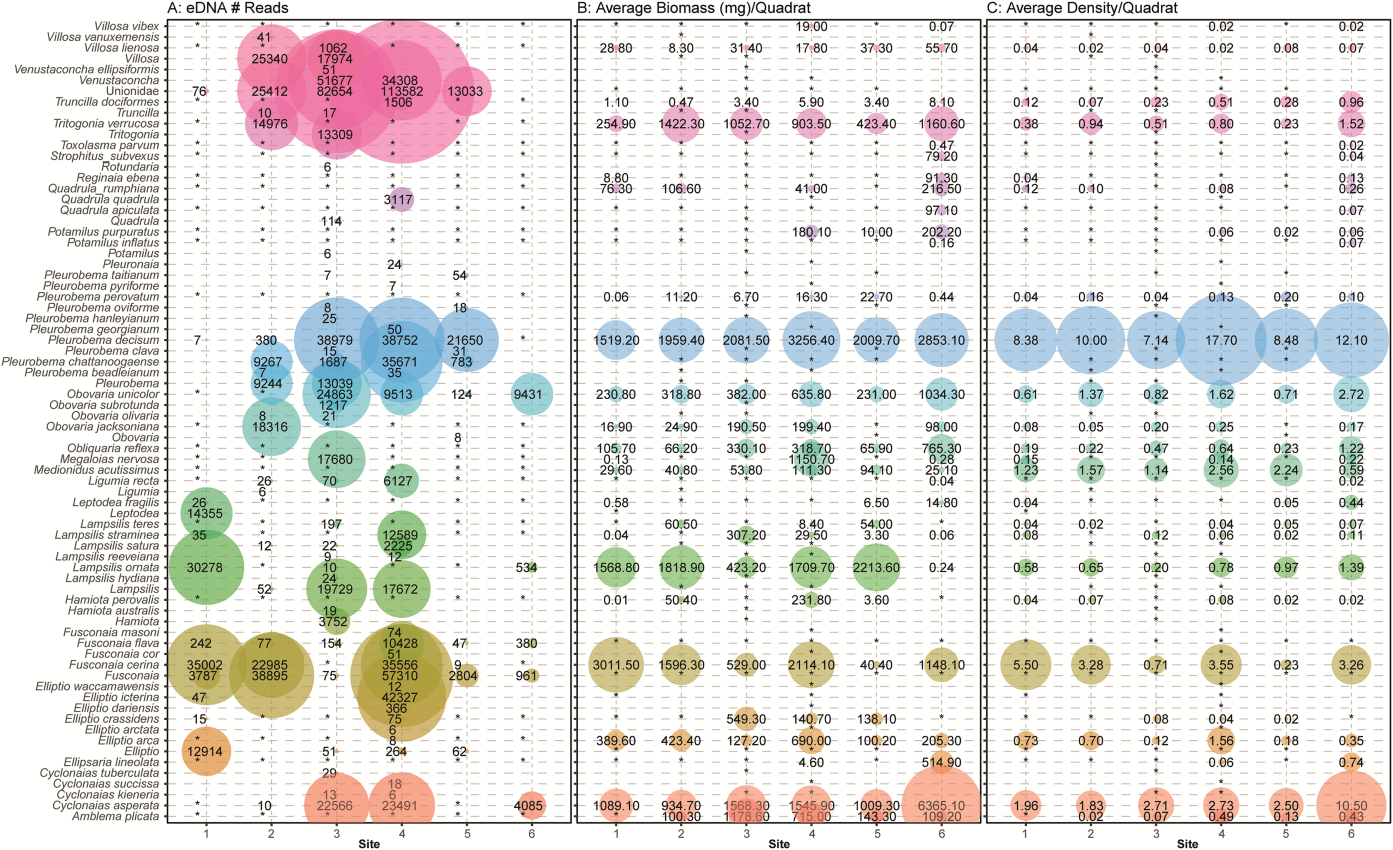
Comparison of eDNA detection of freshwater mussels to field observations from traditional surveys of mussels across six sampling locations in the Sipsey River, Alabama, USA. (A) The total number of reads assigned to classifications using the NCBI database are shown. Field observations for (B) the average biomass of each species in mg/m^2^ and (C) the average density of each mussel species/m2 are shown using taxonomy from Williams et al. (2017). Detections are summed across all sampling times and replicates, with larger bubbles referring to larger metrics, “*” refers to no detection or missing data when comparing eDNA detections with field observations.

In general, the detection of mussel species with eDNA approximated the assemblages recorded by traditional sampling methods with higher eDNA detections for mussel species with larger biomass and greater densities as observed in visual quadrat sampling (Fig. 7). We note, however, that no species had complete congruence across all sites. The species detected at the most sites using eDNA were *Pleurobema decisum* (five out of six sites), *Fusconaia cerina* (four out of six sites), *Obovaria unicolor* (four out of six sites), and *Cyclonaias asperata* (four out of six sites). Similarly, the most abundant species observed in the field surveys were *P. decisum*, *C. asperata*, and *F. cerina*, respectively (Fig. 2). Comparing our traditional samples to eDNA samples, we had 30 unique site/species combinations where the same species were identified by both sampling methods at the same site. Site 4 had the most overlapping species detections with eight species detected by eDNA which is 35% of the 23 observed species (*Elliptio arca, Elliptio crassidens, Fusconaia cerina, Lampsilis straminea, Obovaria unicolor, Pleurobema decisum, Cyclonaias asperata and Truncilla donaciformes*). Site 3 followed with detection of seven overlapping species, 44% of the 16 field observed species (*Lampsilis ornata, Ligumia recta, Megalonaias nervosa, Obovaria unicolor, Pleurobema decisum, Cyclonaias asperata* and *Villosa lienosa)*. We detected six overlapping species at sites 1 (32% of the 19 field observations) and 2 (35% of the 17 field observations) (*Elliptio crassidens, Fusconaia cerina, Lampsilis ornata, Lampsilis straminea, Leptodea fragilis and Pleurobema decisum*) and (*Cyclonais asperata, Fusonaia cerina, Ligumia recta, Obovaria jacksoniana, Pleurobema decisum and Tritogonia verrucosa)* respectively. The sites with the poorest detections were Site 5 and Site 6, which only detected three of the expected species each (*Fusconaia cerina, Obovaria unicolor* and *Pleurobema decisum*) and (*Cyclonaias asperata*, *Lampsilis ornata* and *Obovaria unicolor*) respectively. These detections encompass only 16% of the 19 observed species at Site 5, and 11% of the 27 species observed at Site 6 and are substantially lower than the detection rates from the other four sites.

**Figure 8:**
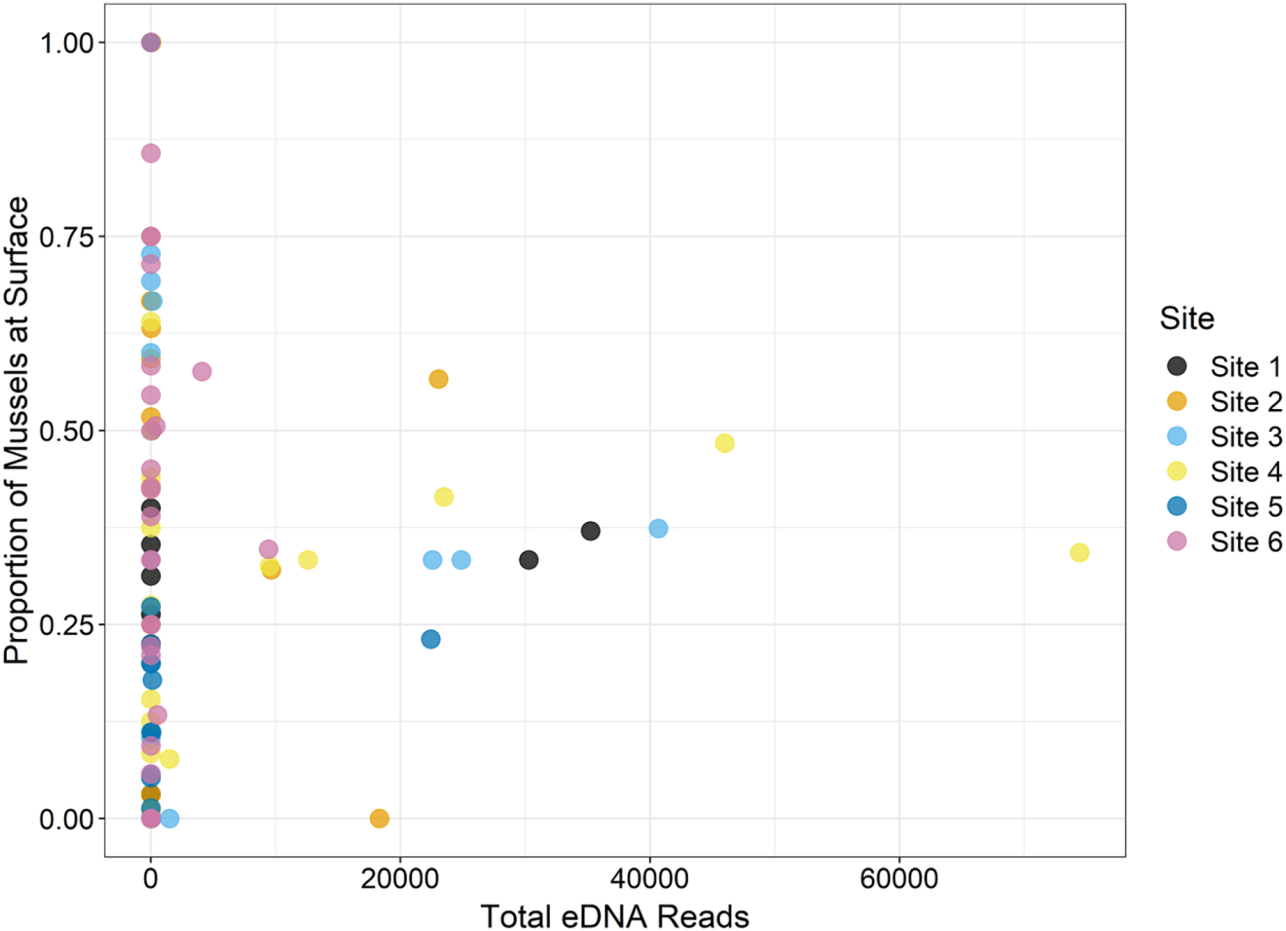
The location of mussels within the substrate, or proportion of mussels at the surface as recorded by traditional surveys, is compared to the total eDNA reads per species across six sampling locations in the Sipsey River, Alabama, USA.

During the traditional survey, we recorded the location of mussels as they were observed (surface *vs*. buried). We hypothesized that a higher number of mussels on the surface would yield greater DNA signal in the sample, but the results are inconsistent and indicate that factors other than density at the surface are influencing species detection (Fig. 8). For example, *Leptodea fragilis* at Site 1 had 100% of the mussels at the surface but only 26 reads counted; however, the average biomass for this mussel at the same site is exceptionally small at 0.58 mg/m^2^. The species with the highest eDNA detections overall, *Pleurobema decisum*, is often buried with an average of ~30% exposed at the surface; however, this species also has the largest average biomass and average density overall. This indicates that biomass and density may influence detection more than substrate location.

Many eDNA detections are classified to the taxonomic level of family (Unionidae) or genus (*e.g*., *Venustaconcha*, *Fusconaia)*. It is possible that some of the missing detections are located at higher taxonomic levels and that insufficient information was captured or available in the database to bioinformatically assign these reads to species. We also noticed that our eDNA classifications returned taxa that may have changed nomenclature per Williams et al. (2017) or are not known to exist in the Sipsey River or the Mobile Basin and were not detected by traditional sampling. This was particularly evident in the *Pleurobemini* tribe containing *Fusconaia* and *Pleurobema* taxonomic groups. For example, within *Fusconaia, F. cerina* was the only species of that genus observed with traditional sampling; however, eDNA detected other *Fusconaia* species with multiple markers (ND1, COI). Of particular note, *F. flava* was detected at all sites with eDNA, but not quadrat sampling. Within *Pleurobema*, the most common species observed was *P. decisum*; however, eDNA data reflected a high number of *P. chattanoogaense* reads at four sites with multiple markers although not mentioned in the traditional survey. While it is possible that traditional methods missed some mussel species or misidentified them, it is more likely that our eDNA classifications generated the discrepancies between quadrat observations and eDNA detections.

Other species detections that were surprising included high numbers of sequences at Site 3 and Site 4 that most closely matched the genus *Venustaconcha*. To the best of our knowledge, no species in that genus has been recorded in the Sipsey. Since the match was not at a close enough% identification to be called at the species level those reads will remain undescribed. We also detected *Lamspsilis satura* and *Elliptio ictarina*, with moderately high numbers of reads at Site 4, and neither species was observed in the field. These may be misclassified detections of other species in that genus that were observed in the field.

### Phylogenetic analysis of pleurobema, fusconaia and eliption species

**Figure 9:**
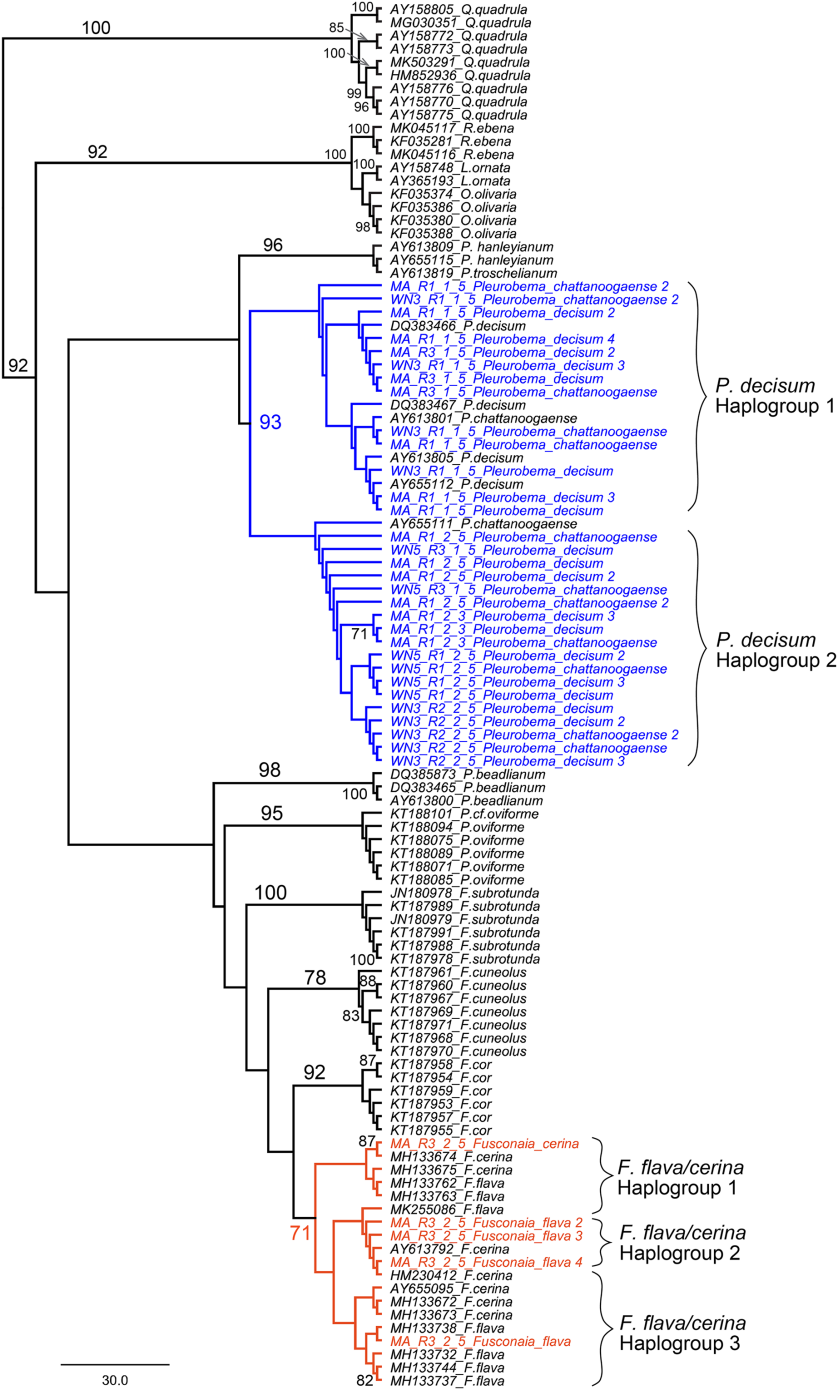
Maximum Likelihood phylogenetic tree containing eDNA sequences collected in the Sipsey River, Alabama, USA. All bootstrap values >70 are shown. Nodes in blue reflect the *Pleurobema decisum* and *P. chattanoogaense* group (our sequences are in blue and the reference sequences in black) and nodes in red reflect the *Fusonaia flava* and *F. cerina group* (our sequences are in red and the reference sequences in black). Brackets contain potential haplogroups captured by this analysis. Tree is rooted with the *Quadrula* group.

We further examined sequences classified to the *Fusconaia, Pleurobema, and Elliptio* taxonomic groups from our ND1 eDNA pool to evaluate congruence between eDNA-based detections and field identification. By adding reference sequences from NCBI to our eDNA sequences, we constructed a maximum likelihood tree that resolves the two prominent *Fusconaia* species in a clade and the two prominent *Pleurobema* species in a clade (Fig. 9).

Our eDNA sequences for *P. decisum* and *P. chattanoogaense* resolved in clades with reference sequences from both species interleaved together with 93% bootstrap support. The resolution of sequences from these two species into two clades indicates that we captured two haplogroups that are represented in both species. The *F. flava* and *F. cerina* sequences also resolved into a single clade, with the reference sequences of both species interleaved together, although with lower bootstrap support (71%). The topology of *Fusonaia* sequences reflect three potential haplogroups, but as with the *Pleurobema* group, these are not well supported by bootstrap values. The mean intraspecific distances for the *Pleurobema* and *Fusonaia* species ranged from 1.15–1.77%; while the interspecific distance between *P. decisum* and *P. chattanoogaense* was 1.84% and between *F. cerina* and *F. flava* was 1.27% (Table 3).

**Table 3:** Summary of genetic distances captured by eDNA detection of ND1 for four *Pleurobemini* species: *Pleurobema decisum, P. chattanoogaense, Fusconaia cerina* and *F. flava*.

Comparison	Average % ID		Std Dev	Range % ID	% Variation
*P.decisum* to *P.decisum*	98.31	+/−	1.06	(95.41–100)	1.69
*P.chatanoogaense* to *P.chatanoogaense*	98.23	+/−	0.91	(95.85–100)	1.77
*P. decisum* to *P. chatanoogaense*	98.16	+/−	1.01	(95.35–100)	1.84
*F. cerina* to *F. cerina*	98.47	+/−	1.42	(95.75–100)	1.53
*F. flava* to *F. flava*	98.85	+/−	0.56	(97.98–100)	1.15
*F. cerina* to *F. flava*	98.73	+/−	0.96	(95.75–100)	1.27
*P. decisum* to *F. flava*	91.39	+/−	1.07	(88.89–93.90)	8.61
*P. decisum* to *F.cerina*	90.35	+/−	2.31	(82.47–93.90)	9.65
*P. chatanoogaense* to *F.cerina*	90.58	+/−	2.32	(82.83–93.81)	9.42
*P. chatanoogaense* to *F.flava*	91.67	+/−	1.02	(89.83–93.33)	8.33

We also generated alignments and maximum likelihood trees using consensus sequences identified as *Elliptio sp*. from our data. There was insufficient resolution at the locus we amplified to identify those reads to the level of species. Our amplified sequences did not group with *E. arca* or *E. crassidens* (both of which was observed in the field), or with any other closely related *Elliptio* species with sequences available in the NCBI reference database. Instead, they formed a separate clade closest to *E. arctata* which has not been seen in the Sipsey for many years. This indicates that the Sipsey River taxon represented by our eDNA sequences is not represented by a sequence in NCBI for an identification match. The failure to match *E. crassidens* or *E. arca* from NCBI also indicates that (a) eDNA primarily sampled another *Elliptio* species besides *E. crassidens* or *E. arca*, (b) *E. crassidens* or *E. arca* has greater haplotype diversity than is represented by NCBI, or (c) that there is an *Elliptio sp*. we detected in the Sipsey River that is a different taxon than those observed in the field study.

## Discussion

Our objective was to evaluate the use of eDNA as a sampling technique for a well-studied assemblage of freshwater mussels in the biologically diverse Sipsey River system in the southeastern USA. Four recent studies have demonstrated the effectiveness of metabarcoding to identify freshwater mussel species in France, Switzerland, Italy, Morocco (Prié et al., 2021), Virginia, USA (Klymus et al., 2021), Ontario, Canada (Coghlan et al., 2021), and Ohio, USA (Marshall et al., 2022). However, these studies describe a range of agreement of species detection from eDNA compared with traditional surveys ranging from 40% to 58% agreement in Virginia/Tennessee (Klymus et al., 2021) to 92% in Ohio (Marshall et al., 2022). Similarly, our eDNA results were broadly consistent with the data from traditional quadrat-based field surveys, although both community eDNA and conventional sampling detected some species that the other method did not. The modifications to the eDNA sampling protocol due to complications with turbid water (use of larger than typical pore filters of a different chemistry) likely explains the low yields of eDNA during extraction and likely contributed to lower species detections overall. Our research project highlighted several continuing challenges with implementing eDNA techniques for quantifying freshwater species assemblages in biologically diverse and turbid rivers.

For those species that were detected in low numbers by eDNA but not by traditional survey, a random sequencing error may have generated the unexpected classification. This explanation is less likely at sites where we have thousands of reads from more than one gene marker indicating an unexpected species is present. For example, we detected *Fusconaia flava* at Site 4 with 10,428 reads from primers targeting ND1 (232) and 16S (10,196). Detecting a species with multiple markers validates that detection. For these instances, it is possible that our eDNA classifications were skewed by very closely related species that share common genetic information within our targeted genes, or that some of the species we detected, and the species observed in the field, are indistinguishable from one another within the gene fragments that we amplified and warrant further investigation. Finally, incorrectly annotated genetic database entries can also contribute to missing detections and challenging interpretations of species identifications.

Our phylogenetic analysis that resolved the two prominent *Fusconaia* species into one clade and the two prominent *Pleurobema* species into one clade may indicate we are capturing a single species for each genus rather than two species each. This is consistent with the conclusions of Campbell et al. (2008) that *P. decisum* and *P. chattanoogaense* are the same species, and that *P. chattanoogaense* as a synonym for *P. decisum* (Williams et al., 2017). These taxa are still listed as separate species in the NCBI database, making their detection by eDNA less straightforward when based on NCBI sequences and classifications. Our findings within *Fusconaia* are less conclusive, given the smaller bootstrap value supporting the resolution of *F. flava* and *F. cerina* in a single clade. Burdick & White (2007) also reported a very close phylogenetic affinity and minimal genetic divergence between these *Fusconaia* species and proposed further study of the taxonomic distinctiveness of *F. cerina* and *F. flava*. Based on our phylogenetic analysis of eDNA sequences classified to the *Pleurobemini* tribe, we suggest that eDNA reads from the Sipsey River classified as *P. chattanoogaense* should be considered as derived from *P. decisum* in the field, and all *F. flava* eDNA sequences should be considered as derived from *F. cerina*. We were unable to determine which *Elliptio* species was detected by eDNA in the Sipsey River, but our field surveys detected both *Elliptio arca* and *E. crassidens*.

Together, our results suggest that taxonomic distinctions for freshwater mussels based on morphology and distribution may be unsupported (and possibly incongruent) by a molecular based approach based on single linked genetic markers like the mitochondrion. Campbell et al. (2008) reported the full length ND1 sequence variation between *P. decisum* and *P. chattanoogaense* to be ~1.07%. This is smaller than the 1.84% that we observed between these species. This may be due to the smaller regions we amplified, which were selected because they show the greatest variation to facilitate metabarcoding identification. All of our fragments for the *Fusconaia* reads were smaller fragments in a region that had less variation from the references. Sometimes they exactly matched the reference or even each other (though each sequence was from different samples). This may have contributed to the lower variation that we observed within that sequence set. Both of these observations emphasize the importance of gene targeting strategies for eDNA detection of closely related species.

Some detection issues may have resulted from mussel life history characteristics and habitat conditions in the Sipsey River. For example, the size, abundance and location in the substrate of mussels may have affected the amount of eDNA detected (Figs. 7 and 8). Availability of mussel DNA is dependent on factors such as the timing of free-floating larval stage and gamete release in conjunction with our eDNA collection times (Schmidt et al., 2013). Many environmental factors can increase the rate of DNA degradation, including water temperature, UV exposure, and microbial communities (reviewed by Coble et al., 2019), all of which may have contributed to our low DNA concentrations. The low DNA yields that led to less than optimal eDNA submission for the array may also have decreased our ability to detect some species that were present, and shed little DNA into the water column (Fig. 5). In addition to increasing species detections, higher DNA yields would have prevented the need to collapse subsamples across sampling periods within a site. We intended to use our repeated sampling design for eDNA in a multi-species occupancy framework, but low yields of DNA prevented this analysis (Dorazio & Royle, 2005; Fukaya et al., 2022).

Collecting and filtering of water samples in slow-moving, turbid, and warm water presented logistical challenges for minimizing DNA degradation. Whereas pumps to filter water in the field work well in many systems with low turbidity, our study required an alternative protocol. Water samples were collected, stored on ice, and then transported to a lab where a vacuum manifold system was used with larger pore size filters to capture as much DNA as possible, but within 24 h of collection to prevent DNA degradation. Innovative approaches to optimize DNA yields in turbid waters is a critical priority for future research. It might be possible to obtain greater yields from turbid water with a sequential filtering strategy by passing water through filters of decreasing pore size, which has been demonstrated as an effective method to overcome volume limitations while reducing pore size in recent qPCR studies (*e.g*., Turner et al., 2014; Moushomi et al., 2019; Cooper et al., 2021). Among multispecies mussel eDNA studies, a range of water volumes have been sampled, typically targeting 0.5 to 1 L (Coghlan et al., 2021; Marshall et al., 2022, this study), but range from 0.045 to 30 L (Klymus et al., 2021; Prié et al., 2021). Optimizing sample volume and filter pore sizes will be necessary for improved multi species eDNA detection in turbid waters.

Additionally, we detected a large number of fish species that are found in the Sipsey River with traditional surveys, mostly of which came from our universal 12S teleost metabarcoding primer. We also detected some fish that were not observed by traditional methods before including *Perca flavescens*, *Ictiobus cyprinellus*, and *Notorus munitus*, and *N. stigmosus*. To better grasp the impact of fishes that host mussel larvae, it may be a good strategy to target specific fish that are important in the life history of mussels. Many of our detections were at the family or genus level as a result of the universal primers that amplified them. Markers that show better resolution, such as ND1 or COI, that directly target certain species would likely provide better resolution into species identity (*e.g*., Hauck et al., 2019).

## Conclusions

With 65% of freshwater mussels considered imperiled and at risk of extinction, the need for non-invasive and reproducible survey methods is greater than ever (Haag & Williams, 2013). Environmental DNA metabarcoding has the potential to reveal a tremendous amount of information for monitoring mussel populations and contributing to conservation options. With a multi-species approach managers can develop information on the mussel fish-host populations, co-occurring aquatic insects, mussel populations present, and the presence of invasive species with a single assay. Future research using metabarcoding eDNA assays for aquatic taxa in the southeastern USA should consider improvements to both sampling logistics and mussel phylogeny. Mussel nomenclature and systematic taxonomy is changing rapidly, with mussel phylogeny derived from molecular genetic data aiding improvements in our understanding of this diverse group of molluscs (Williams et al., 2017). For eDNA metabarcoding to become a more useful tool for monitoring this endangered group of organisms, a full overhaul of all species names attached to those sequences already submitted to NCBI needs to occur. Confidence in our ability to identify eDNA metabarcoding reads relies on a curated, accurate database. Our phylogenetic analysis revealed that substantial corrections are needed to optimize bioinformatic identification of eDNA assays targeting unionid mussels. Improved methodology to enhance eDNA detection coupled with a well curated taxonomic database will then facilitate the use of eDNA to understand environmental effects on mussel assemblages across seasons and years.

## Supplemental Information

10.7717/peerj.15127/supp-1 Supplemental Information 1 Primers developed and used for the Fluidigm Amplification of eDNA samples.

10.7717/peerj.15127/supp-2 Supplemental Information 2 Amplicon templates used to make the positive control.

10.7717/peerj.15127/supp-3 Supplemental Information 3 Interactive Krona chart containing all classified reads from eDNA collected on six sites along the Sipsey River, Alabama, USA.

## Additional Information and Declarations

### Field Study Permissions

The following information was supplied relating to field study approvals (*i.e*., approving body and any reference numbers): Freshwater mussel collection was conducted under US Fish and Wildlife Service permit #TE68616B-1 and Alabama Department of Conservation and Natural Resources permit #2016077745468680 to C. L. Atkinson.

### Data Availability

The following information was supplied regarding data availability: Raw Illumina sequence reads, are available at NCBI: BioProject accession number PRJNA902311.
