# Supplementary material for: Molecular identity crisis: environmental DNA metabarcoding meets traditional taxonomy—assessing biodiversity and freshwater mussel populations (Unionidae) in Alabama
Source: PeerJ. 2023 Apr 3;11:e15127. doi: 10.7717/peerj.15127 (PMC10078462; doi:10.7717/peerj.15127)
Supplement: Supplemental Information 1 [file peerj-11-15127-s001.pdf]

Primer Pair Name	Target Species	Gene Target	Amplicon Length (no Adapters)	Forward Primer	Reverse Primer	Multiplex (third) Primer (F- is forward R- is reverse)
AllAmph12sRana_100+60	All Ranid frogs	12S	337	CAGTCTGTATACCTCCGTCGAAAGCT	CCAGTACACTTACCATGTTACGACTTGC	none
AllAmph12SSal_101+61	All salamanders	12S	406	CCCVCCTAGAGGAGCCTGTTCT	CCTTCCGGTARRCTTACCATGTTACG	none
AllTeleost12s_63+64	All teleost fish	12S	240	GTCAGGTCGAGGTGTAGCGC	CATGTTACGACTTGCCTCCCCT	none
AllChondrostei16S_116+117	All chondrostei fish	16S	335	ACGAGGGCTCAACTGTCTCCT	CCTAGGGTAACTTGGTTCGTTGATCAG	none
AllPhytophthCytB_92+93	*Phytophthora* sp.	COI	261	GCATTTGGTATTATTAGTCAAGTTTCWGCAGC	AGGTGTTTCAAATTTTAAWGAACCRCCCC	none
UnivFlies16S_194+195	Many Aquatic Insects	16S	347	CCGTGCAAAGGTAGCATAATCATTAGTCT	AGAACTCTCRAAAAAGATTACGCTGTTATCCC	none
UnivCrayfish16S_78+191	All Crayfish	16S	302	GGCTAGAATGAATGGTTGGACAAGAAATAATCTG	AGAACTCTYAAAGAAAATTACGCTGTTATCCC	none
SaprolegniaCOI_134+135	*Saprolegnia* sp.	COI	353	GGTACTTTATATATGATTTTTGGTGCTTTTTCAGGT	GGTGGATATACAGTCCAACCAGTACC	none
BdChytridITS_94+95	*Batrachochytrium dendrobatidis*	ITS	176	GGATCTCTTGGCTCTCGCAACG	CATGGTTCATATCTGTCCAGTCAATTCG	none
WhiteNoseCOI_174+175	*Pseudogymnoascus destructans*	COI	290	GGTATATGCGATGATGTCTATTGGAGTTTTAGG	TGTCATGGAAAGCGATATCAAGAGAAGC	none
AllSalamanders2018-12S_198+199	All salamanders	12S	399	AGCTTAAAAYTCAAAGGACTTGRCGG	GRCTTACCWTGTTACGACTTTCCTYTTCT	none
AllSEMussels_16S 200+201	All SE Mussels	16S	214	GHAGACGRAAAGACCCCGCG	GTCGCAAACCCAGCTTTCGAT	none
OuniOjac_COI_Hickorynut 203+204	*Obovaria unicolor, O. jacksoniana and Lampsilis ornata*	COI	213	CTTATRATTGGGGCTCCTGATATGGCT	AGCCCAYACRAACAACGGAATCC	none
Ouni_ND2_Hickorynut 205+206	*Obovaria unicolor*	ND2	203	TACCAGCTTCCTCAAACTACCTACCC	AGAAATGGTTTGGGCTATGGCTCG	none
Lorn_ND1_Pocketbook 209+210	*Lampsilis ornata*	ND1	290	TGAACGCAAAGCCCTCGGG	CTGTTATGAGGGTTGTGTAGACGGC	none
Earc_COI_spike 211+212	*Elliptio arca and E. arctata*	COI	210	TAATGTGATTGTGACGGCACATGC	CCCAACGCCCCTCTCCAC	none
Earc_ND1_spike 213+214	*Elliptio arca and E. arctata*	ND1	142	CCATTCGAGCCATAGCCCAAACC	GCGGAGAGGGCAAGGGTAGG	none
Pdec_COI_SoClubshell 215+216	*Pleurobema decisum*	COI	266	TTGTGCCTGCTCTCTTTTTGTTATTGAGG	CCGCTGTTACCGTCACAGCC	none
PdecPper_ND1_Clubshell 217+218	*Pleurobema decisum and P. perovatum*	ND1	296	TGAACGCAAAGCTTTAGGGTACTTCC	TCAACCTGCTATTAAAGTTGTGTAGACGG	none
Pper_COI_OvateClub 219+220	*Plerobema* sp.	COI	351	GCTTATTCCTCTTATGATTGGGGCTCC	GCAKCAACCAACAAAACCGC	none
Macu_ND1_moccasinshell 221+222	*Medionidus acutissimus*	ND1	262	GAATTCCACAACCATTAGCAGACGC	CCTAAGAGGGCATATTTAGAGTTTGAGGTTC	none
Macu_COI_moccasinshell 223+224	*Medionidus acutissimus*	COI	414	GGCAGCCTGGTAGTTTGTTGG	GCTCAGCAATCAAACCAGGAGATCG	none
Hper_COI_mucket 225+226	*Hamiota perovalis*	COI	429	AACCCAGGAGACCGCATATTCC	GAGTTTGTTAATTCGGGCTGAGCTG	none
LrecLsub_ND1_sandshellpondmussel 227+228	*Ligumia recta* and *L. subrostrata*	ND1	425	GTAAAAGAGTGAGTAATACCAGCCCACT	AATGGRGCYCGATTTGTTTCTGC	none
Lrec_COI_sandshell 229+230	*Ligumia recta*	COI	290	TGACTTATTCCCCTTATGGTTGGAGC	ACTAACCCAGGAGACCGTATATTCCC	none
QaspQver_ND1_OrbPistolgrip 231+232	*Quadrula asperata* and *Q. Verrucosa*	ND1	254	GGRATYCCACAACCATTAGCAGACG	GCGTATTTTGAGTTTGARGCTCAGCC	none
Fcer_COI_Pigtoe 233+234	*Fusconaia cerina*	COI	314	GGTTTTGGTTACTTGTGCCTGCTC	CGGAATTCGCTCAGCAACCA	none
Mner_ND1_Washboard 237+238	*Megalonaias nervosa*	ND1	244	GCCCTCGGAATACTCTTATTTCTGTGC	GGATGGATAGGGTGATGGTAGGTATGG	none
Oref_ND1-Wartyback 239+240	*Obliquaria reflexa*	ND1	257	GGCCCAAATAAAGTAGGAATCATTGGC	CATCCTGCTATGAGGGTTGTGTAGAC	none
Cven_COI_BlacktailShiner 241+242	*Cyprinella venusta*	COI	233	AGTCAACCTGGCTCACTTCTAGG	TCAACACCAGAGGAAGCTAGTAGTAAGAG	none
Cven_CytB_BlacktailShiner 243+244	*Cyprinella venusta*	CytB	349	ATGCGAAGTATAGGACGGAGGC	TGTTCTCCCCTACCCTRCTTGG	none
AnatIpun_CytB_Bullhead-Channel 253+254+255	*Ameiurus natalis* and *Ictalurus punctatus*	CytB	233	AAAGAAACTTGAAACATCGGRGTGGTCC	AATGGAAGTAGGAAGTGAAATGCGAAGA	R-AATGGCAGTAGGAAGTGGAATGCG
Micropterus_CytB_Fwd 260+261	*Micropterus salmoides* and *M. punctulatus*	CytB	352	GCAACCGCCTTCTCATCCG	GGGTGAGGGTGGCATTGTC	none
Munitus_Fwd_CytB 256+257	*Noturus munitus*	CytB	171	GGCTCCAACAATCCGACCG	GTTGGCGGGAGTGAAGTTTTCC	none
Munitus_Fwd_ND5 258+259	*Noturus munitus*	ND5	285	CCTTAAACCAAAACTGGGCCTTAAAGCA	GGTACTTGAAGAATCGGTTTAGATGGGG	none
Pnig_CytB_BlackbandedDarter 264+265	*Percina nigrofasciata*	CytB	351	TCAGTGTGATGAAACTTTGGCTCCT	GGTYGCACCCCAGAAAGACATTTG	none
Pbre_COI_CoalDarter 270+271	*Percina brevicauda*	COI	251	CCTGCCCCCCTCGTTCCT	CAGCTCAAACGAATAAAGGGGTCTGG	none
AllSculpins_CytB_CottusSp 273+274	All N. Amererican Cottus	CytB	316	ACGGTGCCTCTTTYTTCTTCATTTGC	GCAATTACGAAGGGGAADAGGAAGTGG	none
LcyaLgulLmacLmeg_CytB_sunfish 245+246+ 247	All lepomis/sunfish sp.	CytB	369	CCGCCGCAACMGTAATTCACC	TCGTTGTTTGGAGGTGTGGAGG	F-GCCGCCACCGTAATTCACCTAC
Ecae_CytB_RainbowDarter 281+282	*Etheostoma caeruleum*	CytB	324	CAGCTTTCTCATCGGTTGCACAC	AGAAGCCGCCTCAAATCCACTG	none
Pann_CytB_WhiteCrappie 285+286	*Pomoxis annularis*	CytB	196	TGCCCCCTCAAACATCTCAGTCTG	CCTCGGCCAATGTGTARGTAGATGC	none
Casp_ND2_CrystalDarter 287+288	*Crystallaria asprella*	ND2	295	TCTAGGCACCACAATTACCTTCGC	GTGGACGGGGGCAAGACC	none
Mtem_CytB_AligatorSnapper 291+292	*Macrochelys temminckii*	CytB	225	GCAATACACTATTCACCAGACATCTCAATAGC	AGTGGCTATCGTTAGTAATAGGAGGATGAC	none
Mtem_COI_AligatorSnapper 293+294	Macrochelys temminckii	COI	297	TTCTACCCCCATCTTTACTACTACTTCTAGC	CAAGTACAGGCAGTGAAAGTAATAATAGGACA	none
SETurtle3_ND4_GnigGgibGconTsele 301+302+303	Many SE Turtle sp. Including: *G. nigrinoda, G. gibbonsi, P. cnocinna, and T. scripta*	ND4	394	ACCCAATGAGCATACACAGGYGC	GAAGGTGTTCTCGTGTATGGGTTGG	R-CCGTGTATGGGTGGGTGGT
SETurtle3_CytB_GnigGgibGconTsele 304+305	*Trachemys scripta elagans, Graptemys nigrinoda,* and *G. gobbonsi*	CytB	335	TGGACGAGGACTTTATTACGGCTC	CTGTTGGGTTGTTTGATCCAGTTTCATG	none
Gnig_COI_BlackKnobbedMap 295+296	*Graptemys nigrinoda*	COI	295	CGGGAATTGACTTGTGCCATTAATAATTGG	TATGGCTGGGGATTTTATGTTAATTACTGTGG	none
Pcon_CytB_RiverCooter 299+300	*Pseudemys concinna*	CytB	334	TGGACGAGGACTTTACTACGGCTC	TGTTGGGTTGTTTGATCYGGTTTCATG	none
