# Supplementary material for: Molecular identity crisis: environmental DNA metabarcoding meets traditional taxonomy—assessing biodiversity and freshwater mussel populations (Unionidae) in Alabama
Source: PeerJ. 2023 Apr 3;11:e15127. doi: 10.7717/peerj.15127 (PMC10078462; doi:10.7717/peerj.15127)
Supplement: Supplemental Information 2 [file peerj-11-15127-s002.pdf]

Amplicon Templates Used in Positive Control		
**Target Species**	**Target Gene**	**Primer Pair**
**METABARCODING TARGETS (Modified Positive)**		
ALL Ranid Frogs	16s	100 + 60
All Salamanders	16s	101 + 61
ALL Bony Fish	16S	63 + 64
All Chondrostei	16S	116 + 117
Phytophthora	CytB	92 + 93
Batrachochytrium dendrobatidis	ITS	94 + 95
ALL Crayfish	16S	77 + 78
Universal Mussel	16S	200+201
**MUSSELS**		
Obovaria unicolor, Obovaria jacksoniana and Lampsilis ornata	COI	203+204
Obovaria unicolor	ND2	205+206
Obovaria jacksoniana	ND2	207+208
Lampsilis ornata	ND2	209+210
Elliptio arca and Elliptio arctata	COI	211+212
Elliptio arca and Elliptio arctata	ND1	213+214
Pleurobema decisum	COI	215+216
Pleurobema decisum and P. perovatum	ND1	217+218
Plerobema perovatum	COI	219+220
Medionidus acutissimus	ND1	221+222
Medionidus acutissimus	COI	223+224
Hamiota perovalis	COI	225+226
Ligumia recta and Ligumia subrostrata	ND1	227+228
Ligumia recta	COI	229+230
Quadrula asperata and Quadrula verrucosa	ND1	231+232
Fusconaia cerina	COI	233+234
Fusconaia cerina	ND1	235+236
Megalonaias nervosa	ND1	237+238
Obliquaria reflexa	ND1	239+240
**FISH**		
Cyprinella venusta	COI	241+242
Cyprinella venusta	CytB	243+244
Lepomis cyanellus, Lepomis gulosus, Lepomis macrochirus	COI	245+246+ 247
Ameiurus natalis and Ictalurus punctatus (all three primers run together)	CytB	253+254+255
Noturus munitus	CytB	256+257
Noturus munitus	ND5	258+259
Micropterus salmoides and M. punctulatus	CytB	260+261
Micropterus salmoides and M. punctulatus	COI	262+263
Percina nigrofasciata	CytB	264+265
Percina brevicauda	ND2	268+269
Percina brevicauda	COI	270+271
Cottus carolinae	CytB	273+274
Etheostoma caeruleum	CytB	281+282
Pomoxis annularis	CytB	285+286
Crystallaria asprella	ND2	287+288
Crystallaria asprella	COI	289+290
**TURTLES**		
Macrochelys temminckii	CytB	291+292
Macrochelys temminckii	COI	293+294
*****SE Graptemys and Trachymys Turtles:** nigrinoda, gibbonsi, concinna, and scripta elegans*	CytB	304+305
Graptemys nigrinoda and G. gibbonsi	COI	295+296
Pseudemys concinna	CytB	299+300
Sternotherus odoratus and S. carinatus	CytB	306+307
