# Supplementary material for: Molecular identity crisis: environmental DNA metabarcoding meets traditional taxonomy—assessing biodiversity and freshwater mussel populations (Unionidae) in Alabama
Source: PeerJ. 2023 Apr 3;11:e15127. doi: 10.7717/peerj.15127 (PMC10078462; doi:10.7717/peerj.15127)
Supplement: Supplemental Information 3 [file peerj-11-15127-s003.html]

Javascript must be enabled to view this page.

magnitude

All Sites Combined
Site 1 Wendell 6
Site 2 Wendell 5
Site 3 Wendell 3
Site 4 Mussel Mania
Site 5 Wendell 2
Site 6 Station 5

 1677512
 185920
 202148
 435930
 601496
 106136
 145882

 1677489
 185897
 202148
 435930
 601496
 106136
 145882

 1505855
 162368
 182393
 380875
 588230
 88015
 103974

 420111
 65584
 17267
 64521
 141550
 44562
 86627

 402493
 65584
 17267
 64521
 123932
 44562
 86627

 99919
 9618
 1333
 32821
 52307
 75
 3765

 99919
 9618
 1333
 32821
 52307
 75
 3765

 53224
 2590
 1333
 26330
 22786
 0
 185

 205
 0
 0
 0
 205

 24847
 2136
 1270
 9306
 12135

 576
 0
 63
 0
 513

 178
 105
 0
 73

 75
 0
 0
 0
 0
 75

 75
 0
 0
 0
 0
 75

 5539
 0
 0
 5539

 5539
 0
 0
 5539

 177591
 36694
 15922
 3324
 28874
 42853
 49924

 106569
 4765
 0
 0
 10482
 42853
 48469

 45822
 0
 0
 0
 10482
 14335
 21005

 45664
 0
 0
 0
 10482
 14183
 20999

 152
 0
 0
 0
 0
 152

 10
 0
 0
 0
 0
 0
 10

 10
 0
 0
 0
 0
 0
 10

 10
 0
 0
 0
 0
 0
 10

 10
 0
 0
 0
 0
 0
 10

 631
 0
 0
 0
 0
 0
 631

 631
 0
 0
 0
 0
 0
 631

 41
 0
 0
 0
 0
 0
 41

 41
 0
 0
 0
 0
 0
 41

 31499
 4748
 0
 0
 0
 0
 26751

 4838
 4748
 0
 0
 0
 0
 90

 9
 0
 0
 0
 0
 0
 9

 13
 0
 0
 0
 0
 13

 13
 0
 0
 0
 0
 13

 71022
 31929
 15922
 3324
 18392
 0
 1455

 18
 0
 0
 0
 18

 18
 0
 0
 0
 18

 41804
 21887
 0
 88
 18374
 0
 1455

 6
 0
 0
 0
 6

 19911
 0
 0
 88
 18368
 0
 1455

 21887
 21887

 29200
 10042
 15922
 3236

 3292
 0
 56
 3236

 25780
 9914
 15866

 66933
 10798
 0
 11784
 42717
 1634

 66933
 10798
 0
 11784
 42717
 1634

 54501
 0
 0
 11784
 42717

 54440
 0
 0
 11770
 42670

 61
 0
 0
 14
 47

 1634
 0
 0
 0
 0
 1634

 1634
 0
 0
 0
 0
 1634

 10798
 10798

 10798
 10798

 79
 0
 12
 7
 34
 0
 26

 73
 0
 6
 7
 34
 0
 26

 67
 0
 6
 7
 34
 0
 20

 40
 0
 6
 0
 20
 0
 14

 20
 0
 0
 0
 14
 0
 6

 3522
 0
 0
 0
 0
 0
 3522

 3522
 0
 0
 0
 0
 0
 3522

 3522
 0
 0
 0
 0
 0
 3522

 3509
 0
 0
 0
 0
 0
 3509

 13
 0
 0
 0
 0
 0
 13

 42732
 2998
 0
 10344
 0
 0
 29390

 42732
 2998
 0
 10344
 0
 0
 29390

 24
 0
 0
 0
 0
 0
 24

 111
 0
 0
 0
 0
 0
 111

 34
 0
 0
 0
 0
 0
 34

 10344
 0
 0
 10344

 116
 0
 0
 116

 2998
 2998

 2998
 2998

 11717
 5476
 0
 6241

 11717
 5476
 0
 6241

 11717
 5476
 0
 6241

 11717
 5476
 0
 6241

 17618
 0
 0
 0
 17618

 17618
 0
 0
 0
 17618

 17618
 0
 0
 0
 17618

 17618
 0
 0
 0
 17618

 17618
 0
 0
 0
 17618

 1072631
 96784
 165064
 311576
 445193
 38623
 15391

 1072631
 96784
 165064
 311576
 445193
 38623
 15391

 1072631
 96784
 165064
 311576
 445193
 38623
 15391

 1072631
 96784
 165064
 311576
 445193
 38623
 15391

 10549
 0
 10
 29
 6425
 0
 4085

 10502
 0
 10
 0
 6407
 0
 4085

 18
 0
 0
 0
 18

 29
 0
 0
 29

 56147
 12976
 0
 51
 43058
 62

 8
 0
 0
 0
 8

 6
 0
 0
 0
 6

 90
 15
 0
 0
 75

 366
 0
 0
 0
 366

 42374
 47
 0
 0
 42327

 21
 0
 0
 0
 21

 12
 0
 0
 0
 12

 208837
 39031
 61957
 229
 103419
 2860
 1341

 93552
 35002
 22985
 0
 35556
 9

 51
 0
 0
 0
 51

 11328
 242
 77
 154
 10428
 47
 380

 74
 0
 0
 0
 74

 83400
 30313
 64
 19991
 32498
 0
 534

 21
 0
 0
 9
 12

 2259
 0
 12
 22
 2225

 12624
 35
 0
 0
 12589

 30822
 30278
 0
 10
 0
 0
 534

 24
 0
 0
 24

 197
 0
 0
 197

 6229
 0
 32
 70
 6127

 6223
 0
 26
 70
 6127

 63501
 0
 18324
 26101
 9513
 132
 9431

 43931
 0
 0
 24863
 9513
 124
 9431

 29
 0
 8
 21

 1217
 0
 0
 1217

 18316
 0
 18316

 169716
 7
 18898
 53760
 74515
 22536

 13081
 0
 7
 13039
 35

 47408
 0
 9267
 1687
 35671
 783

 99768
 7
 380
 38979
 38752
 21650

 50
 0
 0
 0
 50

 7
 0
 0
 0
 7

 46
 0
 0
 15
 0
 31

 26
 0
 0
 8
 0
 18

 61
 0
 0
 7
 0
 54

 25
 0
 0
 25

 42900
 0
 0
 22693
 20207

 39650
 0
 0
 22566
 17084

 19
 0
 0
 13
 6

 3117
 0
 0
 0
 3117

 1533
 0
 10
 17
 1506

 1506
 0
 0
 0
 1506

 86036
 0
 0
 51728
 34308

 51
 0
 0
 51

 24
 0
 0
 0
 24

 3771
 0
 0
 3771

 19
 0
 0
 19

 437
 0
 0
 437

 437
 0
 0
 437

 17680
 0
 0
 17680

 17680
 0
 0
 17680

 8
 0
 0
 8

 8
 0
 0
 8

 28285
 0
 14976
 13309

 14976
 0
 14976

 44417
 0
 25381
 19036

 1062
 0
 0
 1062

 41
 0
 41

 6
 0
 0
 6

 6
 0
 0
 6

 14381
 14381

 26
 26

 13113
 0
 62
 4778
 1487
 4830
 1956

 12504
 0
 62
 4200
 1487
 4799
 1956

 858
 0
 62
 86
 251
 459

 309
 0
 0
 73
 236

 73
 0
 0
 73

 15
 0
 0
 0
 15

 521
 0
 62
 0
 0
 459

 13
 0
 0
 13

 11646
 0
 0
 4114
 1236
 4340
 1956

 5321
 0
 0
 4085
 1236

 1236
 0
 0
 0
 1236

 32
 0
 0
 0
 32

 1204
 0
 0
 0
 1204

 4085
 0
 0
 4085

 7
 0
 0
 7

 18
 0
 0
 18

 4045
 0
 0
 4045

 7
 0
 0
 7

 8
 0
 0
 8

 6296
 0
 0
 0
 0
 4340
 1956

 29
 0
 0
 29

 496
 0
 0
 465
 0
 31

 31
 0
 0
 0
 0
 31

 31
 0
 0
 0
 0
 31

 465
 0
 0
 465

 66
 0
 0
 66

 66
 0
 0
 66

 66
 0
 0
 66

 66
 0
 0
 66

 66
 0
 0
 66

 47
 0
 0
 47

 47
 0
 0
 47

 47
 0
 0
 47

 47
 0
 0
 47

 140304
 18451
 12185
 43941
 13260
 16695
 35772

 136
 0
 0
 0
 136

 136
 0
 0
 0
 136

 136
 0
 0
 0
 136

 136
 0
 0
 0
 136

 39
 0
 0
 0
 39

 20
 0
 0
 0
 20

 140168
 18451
 12185
 43941
 13124
 16695
 35772

 140168
 18451
 12185
 43941
 13124
 16695
 35772

 140143
 18451
 12166
 43935
 13124
 16695
 35772

 140126
 18451
 12166
 43918
 13124
 16695
 35772

 139254
 18381
 11775
 43649
 13051
 16687
 35711

 66
 0
 0
 34
 32

 28
 0
 0
 6
 6
 16

 81
 0
 0
 15
 34
 32

 44985
 304
 833
 7585
 585
 2175
 33503

 27
 0
 0
 12
 15

 487
 10
 36
 112
 298
 10
 21

 127
 0
 0
 58
 69

 288
 0
 0
 23
 15
 250

 310
 0
 0
 115
 195

 8049
 0
 0
 3186
 4863

 24017
 364
 872
 7063
 6816
 7996
 906

 16
 0
 0
 0
 0
 16

 312
 0
 0
 73
 0
 239

 25
 0
 0
 0
 0
 25

 120
 0
 0
 64
 0
 56

 1208
 0
 0
 1134
 0
 74

 25
 0
 0
 6
 0
 19

 864
 0
 0
 218
 0
 646

 4261
 0
 0
 164
 0
 4097

 2824
 0
 0
 1808
 0
 1016

 219
 0
 0
 219

 10
 0
 0
 10

 14
 0
 0
 14

 6
 0
 0
 6

 25
 0
 0
 25

 21
 0
 0
 21

 6
 0
 0
 6

 8
 0
 0
 8

 32
 0
 0
 32

 28888
 17696
 0
 11192

 7
 7

 6
 0
 0
 0
 6

 8
 0
 0
 0
 0
 8

 225
 0
 0
 225

 225
 0
 0
 225

 25
 0
 19
 6

 25
 0
 19
 6

 6
 0
 0
 6

 26764
 5078
 4154
 10387
 6
 1426
 5713

 26298
 5078
 3688
 10387
 6
 1426
 5713

 26169
 4982
 3688
 10354
 6
 1426
 5713

 26169
 4982
 3688
 10354
 6
 1426
 5713

 16
 0
 10
 0
 6

 26153
 4982
 3678
 10354
 0
 1426
 5713

 129
 96
 0
 33

 129
 96
 0
 33

 129
 96
 0
 33

 466
 0
 466

 466
 0
 466

 466
 0
 466

 4566
 0
 3416
 727
 0
 0
 423

 4566
 0
 3416
 727
 0
 0
 423

 4566
 0
 3416
 727
 0
 0
 423

 4566
 0
 3416
 727
 0
 0
 423

 4566
 0
 3416
 727
 0
 0
 423

 23
 23

 12
 12

 12
 12

 12
 12

 12
 12

 12
 12

 11
 11

 11
 11

 11
 11

 11
 11

 11
 11
